# Student- and School-Level Factors Associated With Mental Health and Well-Being in Early Adolescence

**DOI:** 10.1016/j.jaac.2023.10.004

**Published:** 2024-02

**Authors:** Verena Hinze, Jesus Montero-Marin, Sarah-Jayne Blakemore, Sarah Byford, Tim Dalgleish, Michelle Degli Esposti, Mark T. Greenberg, Benjamin G. Jones, Yasmijn Slaghekke, Obioha C. Ukoumunne, Russell M. Viner, J. Mark G. Williams, Tamsin J. Ford, Willem Kuyken

**Affiliations:** a University of Oxford, Oxford, United Kingdom; b Parc Sanitari Sant Joan de Déu, Sant Boi de Llobregat, Spain, and CIBER of Epidemiology and Public Health (CIBERESP), Madrid, Spain; c Federal University of Pelotas, Pelotas, Brazil; d University of Cambridge, Cambridge, United Kingdom; e Cambridgeshire and Peterborough NHS Foundation Trust, Cambridge, United Kingdom; f University College London, London, United Kingdom; g King’s College London, London, United Kingdom; h Pennsylvania State University, Pennsylvania; i University of Exeter, Exeter, United Kingdom

**Keywords:** adolescence, mental health, multilevel, school, well-being

## Abstract

**Objective:**

Adolescence is a key developmental window that may determine long-term mental health. As schools may influence mental health of students, this study aimed to examine the association of school-level characteristics with students’ mental health over time.

**Method:**

Longitudinal data from a cluster randomized controlled trial comprising 8,376 students (55% female; aged 11-14 years at baseline) across 84 schools in the United Kingdom were analyzed. Data collection started in the academic years 2016/2017 (cohort 1) and 2017/2018 (cohort 2), with follow-up at 1, 1.5, and 2 years. Students’ mental health (risk for depression [Center for Epidemiologic Studies Depression Scale], social-emotional-behavioral difficulties [Strength and Difficulties Questionnaire]) and well-being (Warwick-Edinburgh Mental Well-Being Scale) and relationships with student- and school-level characteristics were explored using multilevel regression models.

**Results:**

Mental health difficulties and poorer well-being increased over time, particularly in girls. Differences among schools represented a small but statistically significant proportion of variation (95% CI) in students’ mental health at each time point: depression, 1.7% (0.9%-2.5%) to 2.5% (1.6%-3.4%); social-emotional-behavioral difficulties, 1.9% (1.1%-2.7%) to 2.8% (2.1%-3.5%); and well-being, 1.8% (0.9%-2.7%) to 2.2% (1.4%-3.0%). Better student-rated school climate analyzed as a time-varying factor at the student and school level was associated with lower risk of depression (regression coefficient [95%CI] student level: −4.25 [−4.48, −4.01]; school level: −4.28 [−5.81, −2.75]), fewer social-emotional-behavioral difficulties (student level: −2.46 [−2.57, −2.35]; school level: −2.36 [−3.08, −1.63]), and higher well-being (student level: 3.88 [3.70, 4.05]; school-level: 4.28 [3.17, 5.38]), which was a stable relationship.

**Conclusion:**

Student-rated school climate predicted mental health in early adolescence. Policy and system interventions that focus on school climate may promote students’ mental health.

Adolescence is a period of rapid social-emotional development that can determine long-term mental health and well-being.^1^^,^^2^ Understanding and targeting vulnerability in adolescence could significantly influence population health as well as educational, occupational, and social outcomes across the life span.

High-quality research considering the role of schools in the mental health of young people is limited. Yet, conceptually, student- and school-level factors could be key to the mental health and well-being of young people, and schools offer an acceptable and efficient opportunity for intervention.^3^ Schools are where most young people spend much of their waking lives. Schools can therefore influence young people’s mental health both directly (eg, management of problems of bullying) and indirectly through aspects of the school community (eg, school ethnicity) or operational features of the school itself (eg, leadership, prevailing culture, and sense of trust or connectedness), which operate within a broader school context (eg, deprivation of its catchment area).^4^, ^5^, ^6^ Research on the role of schools in addressing adolescents’ health and behavioral problems has been active for nearly 50 years. For example, in 1981, the Seattle Social Development Project, a nonrandomized controlled trial combining teacher training with parent education and social competence training for primary school children in urban, high-crime areas in Seattle, was launched.^7^ Students receiving the intervention (vs control) reported less heavy drinking, violent behavior, and sexual intercourse by age 18 years, 6 years after the end of the intervention, underscoring the potential role of schools in promoting students’ health and social adjustment.^7^ More recent research suggested that a positive school climate is associated with a lower risk of not only behavioral but also emotional problems in children and youth; improved student learning, academic achievement, and graduation rates; and higher teacher retention rates.^6^^,^^8^ The growing evidence in this research area has led to government initiatives, such as the Safe and Supportive Schools Project in the United States,^9^ that promote school and home connectedness to reduce students’ risk of physical health (eg, sexually transmitted diseases), mental health (eg, emotional distress), and behavioral (eg, violence and substance use) problems.^9^ Yet, with a strong focus on health and behavioral outcomes, few high-quality studies exist that focus on the long-term relationship between a broad range of school factors and adolescents’ mental health and well-being. Such studies are important to identify the most important factors that could be targeted with universal, school-based interventions.

A recent study of 26,885 11- to 14-year-old adolescents in 85 schools found that schools accounted for a small but statistically significant proportion of the variation in mental health outcomes of students (1.4%-2.4%).^10^ School context (urban area), school community (higher percentage eligible for free school meals), and operational features of the school (school climate rated by teachers) were cross-sectionally associated with students’ mental health and well-being. This study was guided by a theoretical model and highlighted 3 types of school factors that might influence students’ mental health and well-being (Figure S1, available online), including the broader school context (eg, rural/urban location and area-level deprivation), characteristics of the school community (eg, socioeconomic status, level of special educational needs and disability, and ethnic compositions of students within a school), and operational features of the school (eg, school climate, quality ratings, and social-emotional-learning provision as well as school size, student-to-teacher ratio, and mixed-/single-sex schools).^10^ Here, we applied the same theoretical model to explore the influence of a broad range of school factors on students’ mental health over time during early adolescence. First, we estimated the proportion of variation in mental health outcomes of adolescents at the school level at each time point. Second, we explored changes in the associations between student- and school-level factors and mental health outcomes over 2 years. We explored the potential importance of gender differences in all analyses.

## Method

### Study Design and Participants

**Figure 1:**
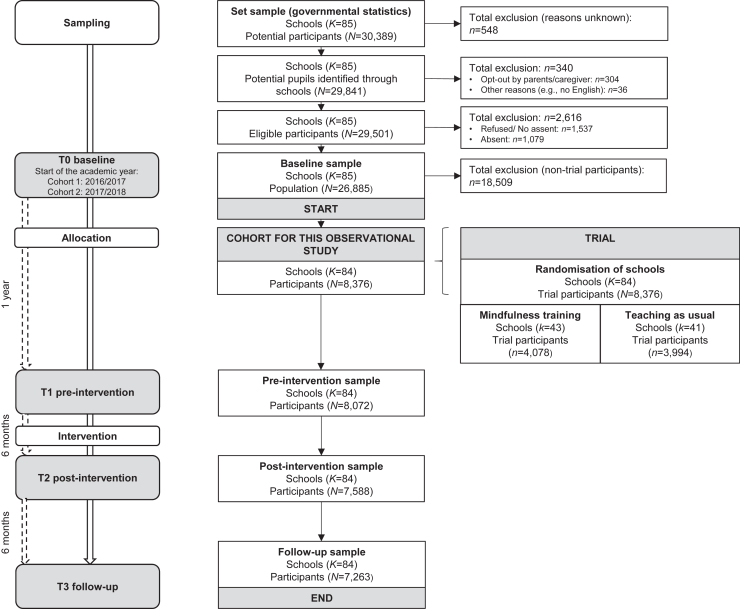
Study Flowchart

This study is a secondary analysis of longitudinal data collected in the MYRIAD (MY Resilience In ADolescence) trial, a 2-arm cluster randomized controlled trial that aimed to establish the effects of school-based mindfulness training on the mental health and well-being of students in early adolescence (ISRCTN86619085, June 3, 2016).^11^^,^^12^ The trial sample consisted of 8,376 students in 84 schools across the United Kingdom (Figure 1). Study participants provided baseline data and were in classes selected for ongoing trial participation. Informed assent/consent was obtained from schools, parents (via opt-out), and students. Participating schools were representative of UK secondary schools (see Supplement 1, available online, for eligibility criteria and representativeness).^10^^,^^13^ The University of Oxford Medical Sciences Division Ethics Committee (R45358/RE001, May 23, 2016) provided ethical approvals for the MYRIAD trial.

Participants were recruited in 2 cohorts. Data collection for the trial sample at baseline (T0) started in the academic years 2016/2017 (cohort 1: n = 975) and 2017/2018 (cohort 2: n = 7,401). Subsequent assessments were carried out after 1 year (preintervention [T1]), 1.5 years (postintervention [T2]), and 2 years (follow-up [T3]) (Figure 1). All data used for the present analyses were collected before the start of the COVID-19 pandemic. There were no differences between school-based mindfulness training (intervention) and teaching as usual in adolescents’ mental health at follow-up in this trial (see Table S1, available online) due to a lack of student engagement.^13^ Hence, we analyzed participants from both trial arms together, but adjusted for trial arm status. Additional information can be found in the trial protocol and update.^11^^,^^12^

### Measures

Student mental health was quantified using 3 outcome measures: risk for depression (Center for Epidemiologic Studies Depression [CES-D] Scale),^14^ with a higher total score (range 0-60) reflecting a greater risk for depression; social-emotional-behavioral difficulties (Strength and Difficulties Questionnaire [SDQ]),^15^ with a higher total score (range 0-40) reflecting more difficulties; and well-being (Warwick-Edinburgh Mental Well-Being Scale [WEMWBS]),^16^^,^^17^ with a higher total score (range 14-70) reflecting greater well-being. Other student-level characteristics included baseline age (analyzed at both the student and the school level), gender (male, female, other/prefer not to say), and ethnicity (White, Asian, Black, and mixed/other ethnic minorities [eg, Arab]).

School-level characteristics refer to the broader school context, school community, and operational school features.^3^^,^^10^ Hence, school-level characteristics are aggregates across students within a school, providing complementary information about the impact of a student’s school environment on their mental health beyond their individual characteristics. Data were obtained by linking publicly available government data to the school’s postal code, unless otherwise specified. We selected measures that were directly comparable across all 4 nations within the United Kingdom (England, Northern Ireland, Scotland, and Wales). Otherwise, we mapped existing measures onto their English equivalent (eg, school quality ratings). The broader school context summarizes wider socioeconomic factors in the school’s catchment area, including urbanicity (urban vs rural school location) and area-level deprivation (Index of Multiple Deprivation, decile rating range 1 [most deprived] to 10 [least deprived]). School community factors refer to characteristics of the student population, including the percentage of students who were eligible for free school meals (school-level economic deprivation), the percentage of students receiving support for special educational needs or disabilities, and students who were White British (all range 0%-100%), as well as the average age of students in a school. Operational school features included the total number of students within a school, student-to-teacher ratio, and school sex (mixed or female-only schools). The most recent official school inspection rating before trial arm allocation was used to obtain an ordinal rating of school quality. As the approach to the measurement of school quality differed in public (independent schools) and private schools and across the nations, we mapped all school inspection rating systems onto the following 3 categories: requires improvements, good, and outstanding. Quality of social-emotional learning (SEL) provision was assessed through a semistructured interview with the senior leadership team or a staff member with overall responsibility for teaching SEL using a list of 16 quality indicators specifically designed for the trial. A higher school rating (range 0-16) reflected better SEL provision.^18^ School attainment was obtained from publicly available government data referring to the average attainment of students within a school.^19^ The attainment score is calculated based on the student’s achievement across 8 subjects, including English and mathematics (double weighted) and 6 further subjects.^20^ The Alaska School Climate and Connectedness Survey (SCCS)^21^ was used to assess teacher-rated (subscales School Leadership and Involvement, Staff Attitudes, and Respectful Climate) and student-rated (subscales School Leadership and Involvement, Respectful Climate, Peer Climate, and Caring Adults) school climate, with higher total scores (range 1-5) representing a better school climate. We conducted confirmatory factor analyses using robust maximum likelihood estimation to confirm that our reduced set of subscales is a valid measure of the underlying school climate concept. Here, school climate was defined as teachers’ and students’ experiences in a school, including feeling safe, connected, and welcomed. It includes connections, partnerships, and conditions for learning (see Supplement 2, available online, for each subscale, the psychometric properties, and relations to students’ mental health over time).^22^ For analysis, overall scores were calculated by taking the mean across teachers (or students for the school-level measure) within a school to obtain a composite school-level measure of teacher-rated (or student-rated) school climate. Student-rated school climate refers to the student’s views of the school climate analyzed both at the student and at the school level. Student-level effects capture each student’s unique perception of the school climate and school-level effects capture the average perception of the school climate among students within a school.

Given the short study period, all student- and school-level factors were measured at baseline (T0) only and assumed to be stable except for student- and teacher-rated school climate, which was considered to be more subjective and changeable and hence was assessed repeatedly. While teacher ratings were available for all time points (T0-T3), student-rated school climate was available only at subsequent assessments (T1-T3) to minimize participant burden at baseline. Additional information on the study measures has been reported elsewhere.^10^

### Statistical Approach

All statistical analyses were performed in R version 3.6.2^23^ (Table S2, available online; for further information, see Supplement 3, available online). We conducted complete case analyses and explored differences between students retained and lost to follow-up. We plotted individual and mean growth curves of adolescents’ mental health over time by gender and prior mental health status (baseline T0 levels) using established cutoff scores.

We estimated intracluster (intraschool) correlation coefficients by fitting 2-level random intercept multilevel linear regressions for each outcome and time point using maximum likelihood estimation (level 1: student level; level 2: school level). The intracluster correlation coefficient shows the extent to which differences in adolescents’ mental health can be explained by differences between schools.^24^ We calculated the 95% CI for the intracluster correlation coefficient based on 100 bootstrap samples.

We analyzed cross-sectional (for school climate factors, assessed repeatedly) and longitudinal (for student- and school-level factors, assessed only at baseline) relationships between student- and school-level factors and mental health outcomes and tested whether there is evidence that those relationships change over time (categorical: 0 [reference], 1 year, 1.5 years, and 2 years). For this, we fitted 3-level random intercept multilevel linear regressions using maximum likelihood estimation (level 1: repeated measurements over time; level 2: student; and level 3: school). All continuous level-2 factors were centered at the school level. All analyses were also stratified by gender.

These analyses were undertaken as follows. First, we examined one factor at a time to explore the effect of each factor on adolescents’ mental health over time. Models with the time by factor interaction term were compared with models with only the respective main effects of time and the factor. If the inclusion of this interaction term provided a better model fit (likelihood ratio test: *p* < .05), then this was used as evidence that the association between the factor and the outcome changes over time. Thus, regression coefficients for the time by factor interaction term reflect changes in the relationship relative to the first assessment. In the absence of statistically significant interactions with time (likelihood ratio test: *p* > .05), we assumed stability in the relationship, and regression coefficients reflect the average relationship across time.

Second, we included statistically significant main or time by factor interaction terms in a series of multivariable models to explore the independent effect of each factor on adolescents’ mental health over time after controlling for all other factors. We adjusted for cohort, trial arm (allocation), and multiple comparisons (Benjamini-Hochberg correction) and estimated 2 models covering 2 years (model 1: T0-T3) or 1 year (model 2: T1-T3), respectively: model 1 comprised student-level demographics + school context + community + operational features except for student-rated school climate, and model 2 comprised all factors in model 1 + student-rated school climate. The absence of baseline student-rated school climate data meant that the first wave (T0) was dropped when student-rated school climate was added as a factor to the multivariable model. Hence, model 2 was considered only to examine the adjusted effects of student-rated school climate on the outcomes.

## Results

### Sample Characteristics

Of the initial sample (N = 8,376 trial participants, K = 84 schools), 7,263 students (86.7%) and all 84 schools were retained until the final time point, and 7,250 students (86.6%) provided data on at least one outcome (Table 1; Table S3, available online). A higher proportion of female students had outcome data at 2-year follow-up compared with male students (55.4% vs 51.1%, *p* = .012). Students with outcome data at 2-year follow-up also reported slightly lower levels of risk of depression (mean [SD] = 13.1 [9.7] vs 15.5 [10.7], *p* < .001) and social-emotional-behavioral difficulties (mean [SD] = 11.5 [6.4] vs 13.5 [6.6], *p* < .001) and higher well-being (mean [SD] = 49.9 [9.6] vs 48.2 [10.2], *p* < .001) at baseline. No other notable differences between students with and without 2-year follow-up data were observed (Table S3, available online). Across time, the percentages of missing data for our outcomes (<2%), time-constant factors (<13.5%) and time-varying factors (teacher-rated school climate: 0%-0.2%; student-rated school climate: 0%-3.4%) were low (Table 1).

**Table 1:** Student (N = 8,376) and School (K = 84) Characteristics Across Time

	T0 (K = 84, N = 8,376; 100%)				T1 (K = 84, n = 8,072; 96.4%)				T2 (K = 84, n = 7,588; 90.6%)				T3 (K = 84, n = 7,263; 86.7%)			
	Value		No. missing	(%)	Value		No. missing	(%)	Value		No. missing	(%)	Value		No. missing	(%)
**Student demographics all measured at baseline (T0)**																
	**Mean**	**(range)**			**Mean**	**(range)**			**Mean**	**(range)**			**Mean**	**(range)**		
Student-level age, in years	12.2	(10.9-14.2)^a^	0	(0)	13.1	(11.9-15.2)	0	(0)	13.7	(12.6-15.8)	0	(0)	14.1	(13.0-16.3)	0	(0)
	**n**	**(%)**			**n**	**(%)**			**n**	**(%)**			**n**	**(%)**		
Gender			156	(1.9)			145	(1.8)			134	(1.8)			131	(1.8)
Female	4,509	(54.9)			4,380	(55.3)			4,126	(55.4)			3,953	(55.4)		
Male	3,547	(43.2)			3,389	(42.8)			3,181	(42.7)			3,044	(42.7)		
Other/prefer not to say	164	(2.0)			158	(2.0)			147	(2.0)			135	(1.9)		
Ethnicity			183	(2.2)			172	(2.1)			159	(2.1)			153	(2.1)
Asian	839	(10.2)			819	(10.4)			785	(10.6)			762	(10.7)		
Black	419	(5.1)			407	(5.2)			365	(4.9)			345	(4.9)		
Mixed and other ethnic minorities	733	(8.9)			707	(8.9)			648	(8.7)			619	(8.7)		
White British	6,202	(75.7)			5,967	(75.5)			5,631	(75.8)			5,384	(75.7)		

**Student mental health measured at each time point**																
	**Mean**	**(SD)**			**Mean**	**(SD)**			**Mean**	**(SD)**			**Mean**	**(SD)**		
Risk of depression, CES-D^b^	13.5	(9.9)	6	(0.1)	15.6	(11.1)	18	(0.2)	16.6	(11.7)	27	(0.4)	16.9	(11.9)	25	(0.3)
	**n**	**(%)**			**n**	**(%)**			**n**	**(%)**			**n**	**(%)**		
Low	5,629	(67.3)			4,735	(58.8)			4,163	(55.1)			3,932	(54.3)		
At risk	1,912	(22.8)			2,100	(26.1)			1,989	(26.3)			1,891	(26.1)		
Caseness	829	(9.9)			1,219	(15.1)			1,409	(18.6)			1,415	(19.5)		
	**Mean**	**(SD)**			**Mean**	**(SD)**			**Mean**	**(SD)**			**Mean**	**(SD)**		
Social-emotional-behavioral difficulties, SDQ^c^	11.8	(6.5)	124	(1.5)	12.4	(6.6)	30	(0.4)	13.3	(6.9)	46	(0.6)	13.1	(6.8)	38	(0.5)
	**n**	**(%)**			**n**	**(%)**			**n**	**(%)**			**n**	**(%)**		
Normal	5,576	(67.6)			5,184	(64.5)			4,481	(59.4)			4,321	(59.8)		
Borderline	1,066	(12.9)			1,022	(12.7)			1,041	(13.8)			1,005	(13.9)		
High	519	(6.3)			572	(7.1)			573	(7.6)			544	(7.5)		
Very high	1,091	(13.2)			1,264	(15.7)			1,447	(19.2)			1,355	(18.8)		
	**Mean**	**(SD)**			**Mean**	**(SD)**			**Mean**	**(SD)**			**Mean**	**(SD)**		
Well-being, WEMWBS^d^	49.7	(9.7)	43	(0.5)	49.1	(9.1)	14	(0.2)	47.9	(9.5)	16	(0.2)	47.6	(9.8)	19	(0.3)
	**n**	**(%)**			**n**	**(%)**			**n**	**(%)**			**n**	**(%)**		
Probable mental health difficulties	1,445	(17.3)			1,371	(17.0)			1,566	(20.7)			1,619	(22.3)		
Possible mental health difficulties	963	(11.6)			967	(12.0)			1,026	(13.5)			981	(13.5)		
Average mental well-being	4,583	(55.0)			4,762	(59.1)			4,219	(55.7)			3,907	(53.9)		
High well-being	1,342	(16.1)			958	(11.9)			761	(10.1)			737	(10.2)		

**School context all measured at baseline (T0)**																
	**k**	**(%)**			**k**	**(%)**			**k**	**(%)**			**k**	**(%)**		
Urbanicity			0	(0)			0	(0)			0	(0)			0	(0)
Rural	13	(15.5)			13	(15.5)			13	(15.5)			13	(15.5)		
Urban	71	(84.5)			71	(84.5)			71	(84.5)			71	(84.5)		
	**Mean**	**(SD)**			**Mean**	**(SD)**			**Mean**	**(SD)**			**Mean**	**(SD)**		
Area-level deprivation, IMD	5.8	(2.7)	0	(0)	5.8	(2.7)	0	(0)	5.8	(2.7)	0	(0)	5.8	(2.7)	0	(0)

**School community all measured at baseline (T0)**																
Percentage of students eligible for free school meals	12.5	(9.4)	0	(0)	12.5	(9.4)	0	(0)	12.5	(9.4)	0	(0)	12.5	(9.4)	0	(0)
Percentage of students receiving SEND support	10.1	(5.5)	6	(0.7)	10.1	(5.5)	6	(0.7)	10.1	(5.5)	6	(0.7)	10.1	(5.5)	6	(0.7)
Percentage of students who are White British	76.9	(24.1)	5	(0.6)	76.9	(24.1)	5	(0.6)	76.9	(24.1)	5	(0.6)	76.9	(24.1)	5	(0.6)
	**Mean**	**(range)**			**Mean**	**(range)**			**Mean**	**(range)**			**Mean**	**(range)**		
School-level age in years	11.7	(11.1-12.3)	0	(0)	11.7	(11.1-12.3)	0	(0)	11.7	(11.1-12.3)	0	(0)	11.7	(11.1-12.3)	0	(0)

**Operational features of the school all measured at baseline (T0)**																
	**Mean**	**(SD)**			**Mean**	**(SD)**			**Mean**	**(SD)**			**Mean**	**(SD)**		
Number of students	1,013.0	(337.8)	0	(0)	1,013.0	(337.8)	0	(0)	1,013.0	(337.8)	0	(0)	1,013.0	(337.8)	0	(0)
	**k**	**(%)**			**k**	**(%)**			**k**	**(%)**			**k**	**(%)**		
Mixed-/single-sex school			0	(0)			0	(0)			0	(0)			0	(0)
Mixed	73	(86.9)			73	(86.9)			73	(86.9)			73	(86.9)		
Female only	11	(13.1)			11	(13.1)			11	(13.1)			11	(13.1)		
	**Mean**	**(SD)**			**Mean**	**(SD)**			**Mean**	**(SD)**			**Mean**	**(SD)**		
Student-to-teacher ratio	15.9	(1.9)	9	(10.7)	15.9	(1.9)	9	(10.7)	15.9	(1.9)	9	(10.7)	15.9	(1.9)	9	(10.7)
	**k**	**(%)**			**k**	**(%)**			**k**	**(%)**			**k**	**(%)**		
School quality			11	(13.1)			11	(13.1)			11	(13.1)			11	(13.1)
Requires improvement	11	(15.1)			11	(15.1)			11	(15.1)			11	(15.1)		
Good	46	(63.0)			46	(63.0)			46	(63.0)			46	(63.0)		
Outstanding	16	(21.9)			16	(21.9)			16	(21.9)			16	(21.9)		
	**Mean**	**(SD)**			**Mean**	**(SD)**			**Mean**	**(SD)**			**Mean**	**(SD)**		
SEL provision quality rating	12.0	(2.6)	0	(0)	12.0	(2.6)	0	(0)	12.0	(2.6)	0	(0)	12.0	(2.6)	0	(0)
School attainment	46.1	(14.5)	10	(11.9)	46.1	(14.5)	10	(11.9)	46.1	(14.5)	10	(11.9)	46.1	(14.5)	10	(11.9)

**Time-varying factors measured at each time point**																
Teacher-rated school climate, SCCS	3.9	(0.3)	0	(0)	3.9	(0.3)	0	(0)	3.8	(0.4)	0	(0)	3.9	(0.3)	2	(0.2)
Student-rated school climate, SCCS																
Student-level	NA		NA		3.5	(0.7)	267	(3.3)	3.3	(0.7)	256	(3.4)	3.3	(0.7)	176	(2.4)
School-level	NA		NA		3.5	(0.2)	0	(0)	3.3	(0.2)	0	(0)	3.3	(0.2)	0	(0)

Note: CES-D = Center for Epidemiological Studies Depression Scale; IMD = Index of multiple deprivation; *K* = number of schools; *N* = number of students; NA = not available; SCCS = School Climate and Connectedness Survey; SDQ = Strengths and Difficulties Questionnaire; SEL = social and emotional learning; SEND = special educational needs and disability; T0 = baseline (0 years), T1 = preintervention (1 year), T2 = postintervention (1.5 years), T3 = follow-up (2 years); WEMWBS = Warwick-Edinburgh Mental Well-Being Scale.

a At baseline, most students were aged 11 (41.9%) or 12 years (49.3%).

b Two cutoff points have previously been proposed to identify students at risk of depression (≥16) and with symptoms likely to meet diagnostic criteria for major depressive disorder (≥28). These 2 validated criteria were used to categorize participants into 3 subgroups for descriptive purposes (low: 0-15; at risk: 16-27; probable caseness: 28-60).

c The validated 4-band categorization was used for descriptive purposes: (normal: 0-14; borderline: 15-17; high: 18-19; very high: 20-24).

d The WEMWBS has been validated in UK population-based samples and benchmarked against validated measures of depression, suggesting cutoff values, which were used for descriptive purposes (probable mental health difficulties: 0-40; possible mental health difficulties: 41-44; average mental well-being: 45-59; high well-being: 60-70).

**Figure 2:**
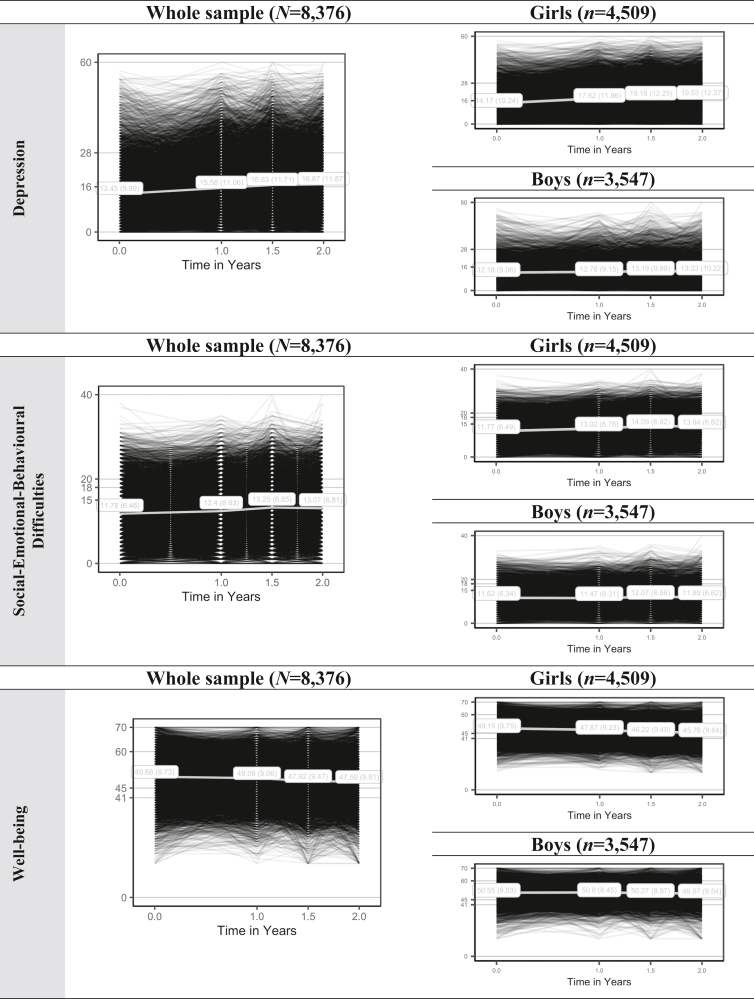
Adolescents’ Mental Health Growth Curves Overall and by Gender ***Note:****(A-I) Cutoff scores are based on the official scoring guidelines. Depression: low (0-15), at risk of depression (16-27), caseness (28-60). Social-emotional-behavioral difficulties: normal (0-14), borderline (15-17), high (18-19), very high (20-40). Well-being: probable mental health difficulties (0-40), possible mental health difficulties (41-44), average mental well-being (45-59), high well-being (60-70). Please note color figures are available online.*

Table 1 shows student and school characteristics across time. Students’ mental health at baseline was within normal ranges, ie, low risk of depressive symptoms (CES-D: mean [SD] = 13.5 [9.9]) and social-emotional-behavioral difficulties (SDQ: mean [SD] = 11.8 [6.5]) and average mental well-being (WEMWBS: mean [SD] = 49.7 [9.7]). At subsequent assessments, mental health worsened, such that students’ rounded mean scores fell within the at-risk range of risk for depression (T1-T3).^14^ This increase was more pronounced in girls than boys (Figure 2). Additional analyses showed that differences in adolescents’ mental health narrowed over time. That is, adolescents with worse baseline mental health typically improved, while adolescents with better baseline mental health tended to decline (Figure S2, available online).

Across all time points, differences between schools accounted for a small yet statistically significant proportion of the total variation in adolescents’ risk for depression (1.7%-2.5%), social-emotional-behavioral difficulties (1.9%-2.8%), and well-being (1.8%-2.2%), which was evident for both genders, particularly girls (Table 2).

**Table 2:** Intracluster Correlation Coefficient (ICC) for School-Level Variance of Students’ Mental Health by Time and Gender

Whole sample																
Students’ mental health	T0				T1				T2				T3			
	Sample size		ICC	[95% CI]	Sample size		ICC	[95% CI]	Sample size		ICC	[95% CI]	Sample size		ICC	[95% CI]
	N	K			N	K			N	K			N	K		
CES-D	8,370	84	0.017	[0.009, 0.025]	8,054	84	0.025	[0.016, 0.034]	7,561	84	0.022	[0.013, 0.031]	7,238	84	0.022	[0.013, 0.031]
SDQ	8,252	84	0.028	[0.021, 0.035]	8,042	84	0.023	[0.015, 0.031]	7,542	84	0.024	[0.015, 0.033]	7,225	84	0.019	[0.011, 0.027]
WEMWBS	8,333	84	0.019	[0.012, 0.026]	8,058	84	0.022	[0.014, 0.030]	7,572	84	0.020	[0.012, 0.028]	7,244	84	0.018	[0.009, 0.027]

Boys																
Students’ mental health	T0				T1				T2				T3			
	Sample size		ICC	[95% CI]	Sample size		ICC	[95% CI]	Sample size		ICC	[95% CI]	Sample size		ICC	[95% CI]
	n	k			n	k			n	k			n	k		
CES-D	3,546	75	0.013	[−0.005, 0.031]	3,378	75	0.009	[−0.006, 0.024]	3,164	75	0.013	[−0.003, 0.029]	3,031	74	0.007	[−0.009, 0.023]
SDQ	3,546	75	0.024	[0.007, 0.041]	3,368	75	0.021	[0.005, 0.037]	3,155	75	0.021	[0.005, 0.037]	3,021	74	0.012	[−0.005, 0.029]
WEMWBS	3,546	75	0.019	[0.004, 0.034]	3,384	75	0.022	[0.005, 0.039]	3,172	75	0.019	[0.004, 0.034]	3,035	74	0.013	[−0.002, 0.028]

Girls																
Students’ mental health	T0				T1				T2				T3			
	Sample size		ICC	[95% CI]	Sample size		ICC	[95% CI]	Sample size		ICC	[95% CI]	Sample size		ICC	[95% CI]
	n	k			n	k			n	k			n	k		
CES-D	4,509	84	0.029	[0.014, 0.044]	4,373	84	0.044	[0.027, 0.061]	4,116	84	0.037	[0.021, 0.053]	3,943	84	0.036	[0.018, 0.054]
SDQ	4,506	84	0.040	[0.025, 0.055]	4,372	84	0.040	[0.023, 0.057]	4,107	84	0.043	[0.027, 0.059]	3,941	84	0.040	[0.021, 0.059]
WEMWBS	4,508	84	0.027	[0.012, 0.042]	4,372	84	0.040	[0.024, 0.056]	4,120	84	0.039	[0.023, 0.055]	3,945	84	0.030	[0.013, 0.047]

Note: ICC refers to the proportion of total variance in the outcome attributed to the school level. Multilevel models are based on complete case analysis. The sample consists of K = 84 schools and N = 8,376 students (boys: n = 3,547; girls: n = 4,509), but the number varies due to missingness. CES-D = Center for Epidemiologic Studies Depression Scale; SDQ = Strengths and Difficulties Questionnaire; T0 = baseline (0 years), T1 = preintervention (1 year), T2 = postintervention (1.5 years), T3 = follow-up (2 years); WEMWBS = Warwick-Edinburgh Mental Well-Being Scale.

### Factors Associated With Changes in Adolescents’ Mental Health

Table 3 shows student- and school-level factors that were associated with changes in adolescents’ mental health over time in fully adjusted, multivariable models (for girls see Table S4, available online; for boys see Table S5, available online). Similar results were obtained for model 1 (T0-T3: 2-year period without student-rated school climate) and model 2 (T1-T3: 1-year period with student-rated school climate), unless noted explicitly.

**Table 3:** Multivariable Analyses (Adjusted for Cohort, Allocation, and Multiple Comparisons) of Repeated Associations Based on the Three-Level Random Intercept Model

	Depression, CES-D			Social-emotional-behavioral difficulties, SDQ			Well-being, WEMWBS		
	Coefficient	95% CI	*p*	Coefficient	95% CI	*p*	Coefficient	95% CI	*p*
**Model 1: Student-level + school context/community/operational features (except student-rated school climate)**									
Age^a^	0.53	[0.16, 0.90]	**.005**∗	0.41	[0.14, 0.67]	**.002**∗	−0.87	[−1.25, −0.50]	**< .001**∗
Time (T1) × age	NA	NA	NA	−0.17	[−0.39, 0.05]	.120	0.08	[−0.28, 0.45]	.650
Time (T2) × age	NA	NA	NA	−0.25	[−0.48, −0.03]	**.027**	0.48	[0.11, 0.86]	**.012**∗
Time (T3) × age	NA	NA	NA	−0.27	[−0.50, −0.04]	**.019**	0.46	[0.08, 0.85]	**.017**
Gender (female)	2.20	[1.64, 2.76]	**< .001**∗	0.40	[0.06, 0.73]	**.020**	−1.75	[−2.22, −1.28]	**< .001**∗
Time (T1) × gender	3.14	[2.61, 3.66]	**< .001**∗	1.41	[1.13, 1.68]	**< .001**∗	−1.60	[−2.06, −1.13]	**< .001**∗
Time (T2) × gender	4.07	[3.54, 4.61]	**< .001**∗	1.84	[1.56, 2.13]	**< .001**∗	−2.77	[−3.24, −2.29]	**< .001**∗
Time (T3) × gender	4.30	[3.75, 4.85]	**< .001**∗	1.93	[1.64, 2.22]	**< .001**∗	−2.88	[−3.37, −2.40]	**< .001**∗
Ethnicity (White)	NA	NA	NA	0.80	[0.45, 1.15]	**< .001**∗	NA	NA	NA
Urbanicity (rural)	−1.17	[−2.25, −0.09]	**.034**	−0.95	[−1.66, −0.23]	**.009**∗	1.09	[0.28, 1.89]	**.008**∗
Percentage of free school meals	NA	NA	NA	−0.01	[−0.04, 0.03]	.769	NA	NA	NA
Time (T1) × meals	NA	NA	NA	0.02	[0.00, 0.04]	**.012**∗	NA	NA	NA
Time (T2) × meals	NA	NA	NA	0.03	[0.01, 0.05]	**.002**∗	NA	NA	NA
Time (T3) × meals	NA	NA	NA	0.04	[0.02, 0.06]	**< .001**∗	NA	NA	NA
Percentage of school ethnicity (= White)	0.01	[0.00, 0.03]	.117	NA	NA	NA	−0.03	[−0.04, −0.01]	**.001**∗
Time (T1) × school ethnicity	−0.01	[−0.02, 0.01]	.404	NA	NA	NA	0.01	[0.00, 0.02]	.198
Time (T2) × school ethnicity	−0.01	[−0.02, 0.01]	.319	NA	NA	NA	0.01	[0.00, 0.02]	.244
Time (T3) × school ethnicity	0.01	[−0.01, 0.02]	.508	NA	NA	NA	0.01	[−0.01, 0.01]	.637
School-level age	0.52	[−1.04, 2.08]	.514	1.11	[0.14, 2.09]	**.024**	NA	NA	NA
Time (T1) × age	1.55	[0.51, 2.60]	**.004**∗	−0.04	[−0.60, 0.53]	.895	NA	NA	NA
Time (T2) × age	0.30	[−0.75, 1.36]	.572	−0.77	[−1.35, −0.20]	**.008**∗	NA	NA	NA
Time (T3) × age	0.11	[−0.96, 1.18]	.843	−0.82	[−1.41, −0.24]	**.005**∗	NA	NA	NA
School size per 100 students	NA	NA	NA	−0.01	[−0.10, 0.07]	.791	NA	NA	NA
Time (T1) × size	NA	NA	NA	−0.05	[−0.09, 0.00]	.060	NA	NA	NA
Time (T2) × size	NA	NA	NA	0.01	[−0.04, 0.06]	.807	NA	NA	NA
Time (T3) × size	NA	NA	NA	−0.02	[−0.07, 0.03]	.431	NA	NA	NA
School sex (female)	−0.35	[−1.83, 1.13]	.643	−0.10	[−1.01, 0.80]	.822	0.19	[−1.00, 1.38]	.751
Time (T1) × sex	−1.16	[−2.15, −0.18]	**.021**	−0.34	[−0.87, 0.19]	.206	0.74	[−0.15, 1.62]	.103
Time (T2) × sex	0.28	[−0.72, 1.27]	.589	0.11	[−0.42, 0.64]	.689	0.29	[−0.60, 1.18]	.518
Time (T3) × sex	−0.07	[−1.08, 0.93]	.886	−0.48	[−1.02, 0.05]	.075	−0.03	[−0.93, 0.87]	.947
School quality (good)^b^	0.03	[−1.24, 1.29]	.967	0.46	[−0.32, 1.25]	.246	−0.89	[−1.86, 0.07]	.069
School quality (requires improvement)^b^	−0.29	[−1.86, 1.29]	.720	0.82	[−0.15, 1.80]	.098	−0.80	[−2.00, 0.40]	.191
Time (T1) × quality (good)	−0.46	[−1.29, 0.37]	.279	−0.32	[−0.76, 0.12]	.154	0.75	[0.04, 1.45]	**.038**
Time (T2) × quality (good)	0.57	[−0.27, 1.41]	.180	0.02	[−0.43, 0.46]	.936	0.53	[−0.19, 1.24]	.148
Time (T3) × quality (good)	0.38	[−0.47, 1.22]	.383	−0.17	[−0.62, 0.28]	.462	0.34	[−0.38, 1.06]	.350
Time (T1) × quality (RI)	−0.43	[−1.50, 0.64]	.431	−0.73	[−1.32, −0.15]	**.014**	1.15	[0.22, 2.07]	**.015**
Time (T2) × quality (RI)	1.03	[−0.09, 2.14]	.070	−0.27	[−0.88, 0.34]	.381	0.60	[−0.36, 1.56]	.223
Time (T3) × quality (RI)	1.41	[0.30, 2.53]	**.013**	−0.21	[−0.82, 0.39]	.490	−0.31	[−1.27, 0.64]	.521
School attainment	−0.03	[−0.08, 0.01]	.121	NA	NA	NA	NA	NA	NA
Time (T1) × attainment	0.04	[0.01, 0.07]	**.011**	NA	NA	NA	NA	NA	NA
Time (T2) × attainment	0.03	[0.00, 0.06]	**.022**	NA	NA	NA	NA	NA	NA
Time (T3) × attainment	0.03	[0.00, 0.06]	**.040**	NA	NA	NA	NA	NA	NA
Time-varying factor									
School climate (teacher)	−0.18	[−1.21, 0.85]	.728	−0.37	[−0.92, 0.18]	.190	−0.21	[−1.09, 0.67]	.645
Time (T1) × climate	−0.71	[−1.79, 0.37]	.200	0.17	[−0.39, 0.73]	.551	0.46	[−0.45, 1.38]	.320
Time (T2) × climate	−0.06	[−1.14, 1.03]	.917	0.21	[−0.36, 0.78]	.475	−0.03	[−0.96, 0.90]	.949
Time (T3) × climate	−0.34	[−1.44, 0.75]	.537	0.41	[−0.16, 0.99]	.158	0.32	[−0.61, 1.26]	.498
**Model 2: Student-level + school context/community/operational features (including student-rated school climate)**									
Age^a^	0.26	[−0.12, 0.64]	.177	−0.06	[−0.31, 0.19]	.644	−0.38	[−0.73, −0.03]	**.033**
Time (T2) × age	NA	NA	NA	0.04	[−0.16, 0.25]	.671	0.18	[−0.17, 0.52]	.313
Time (T3) × age	NA	NA	NA	0.11	[−0.10, 0.31]	.308	0.15	[−0.20, 0.50]	.397
Gender (female)	5.23	[4.67, 5.78]	**< .001**∗	1.70	[1.39, 2.02]	**< .001**∗	−3.21	[−3.65, −2.77]	**< .001**∗
Time (T2) × gender	0.73	[0.22, 1.23]	**.005**∗	0.43	[0.17, 0.69]	**.001**∗	−1.00	[−1.44, −0.57]	**< .001**∗
Time (T3) × gender	0.97	[0.45, 1.50]	**< .001**∗	0.51	[0.25, 0.77]	**< .001**∗	−1.20	[−1.64, −0.76]	**< .001**∗
Ethnicity (White)	NA	NA	NA	0.95	[0.61, 1.29]	**< .001**∗	NA	NA	NA
Urbanicity (rural)	−1.19	[−2.28, −0.10]	**.032**	−0.94	[−1.57, −0.32]	**.003**∗	0.92	[0.18, 1.66]	**.014**∗
Percentage of free school meals	NA	NA	NA	0.02	[−0.01, 0.05]	.219	NA	NA	NA
Time (T2) × meals	NA	NA	NA	0.01	[−0.01, 0.02]	.855	NA	NA	NA
Time (T3) × meals	NA	NA	NA	0.01	[−0.01, 0.02]	.367	NA	NA	NA
Percentage of school ethnicity (= White)	0.02	[0.00, 0.04]	.066	NA	NA	NA	−0.02	[−0.04, −0.01]	**.001**∗
Time (T2) × school ethnicity	−0.01	[−0.02, 0.01]	.438	NA	NA	NA	0.01	[−0.01, 0.01]	.854
Time (T3) × school ethnicity	0.01	[0.00, 0.02]	.229	NA	NA	NA	0.01	[−0.01, 0.01]	.981
School-level age	1.07	[−0.53, 2.67]	.190	0.67	[−0.21, 1.54]	.136	NA	NA	NA
Time (T2) × age	−1.02	[−2.02, −0.01]	**.048**	−0.70	[−1.22, −0.17]	**.009**∗	NA	NA	NA
Time (T3) × age	−1.23	[−2.27, −0.19]	**.020**	−0.68	[−1.21, −0.14]	**.013**∗	NA	NA	NA
School size (per 100 students)	NA	NA	NA	−0.03	[−0.11, 0.04]	.401	NA	NA	NA
Time (T2) × size	NA	NA	NA	0.05	[0.00, 0.09]	**.047**	NA	NA	NA
Time (T3) × size	NA	NA	NA	0.01	[−0.04, 0.06]	.657	NA	NA	NA
School sex (female)	−1.56	[−3.02, −0.09]	.037	−0.34	[−1.14, 0.46]	.409	0.90	[−0.17, 1.98]	.100
Time (T2) × sex	1.10	[0.17, 2.04]	**.020**	0.26	[−0.22, 0.74]	.284	−0.22	[−1.03, 0.58]	.587
Time (T3) × sex	0.80	[−0.15, 1.75]	.097	−0.33	[−0.81, 0.16]	.188	−0.48	[−1.30, 0.33]	.245
School quality (good)^b^	−0.46	[−1.69, 0.78]	.469	0.12	[−0.57, 0.80]	.739	−0.09	[−0.94, 0.77]	.842
School quality (requires improvement)^b^	−0.68	[−2.22, 0.85]	.384	−0.03	[−0.89, 0.82]	.937	0.40	[−0.67, 1.47]	.465
Time (T2) × quality (good)	0.83	[0.06, 1.59]	**.034**	0.19	[−0.20, 0.58]	.334	0.04	[−0.59, 0.66]	.910
Time (T3) × quality (good)	0.69	[−0.09, 1.47]	.083	−0.01	[−0.40, 0.39]	.982	−0.16	[−0.79, 0.46]	.608
Time (T2) × quality (RI)	1.04	[0.00, 2.07]	**.049**	0.24	[−0.30, 0.78]	.393	−0.25	[−1.10, 0.60]	.567
Time (T3) × quality (RI)	1.82	[0.77, 2.87]	**.001**∗	0.44	[−0.10, 0.97]	.113	−1.30	[−2.15, −0.46]	**.003**∗
School attainment	0.02	[−0.02, 0.06]	.361	NA	NA	NA	NA	NA	NA
Time (T2) × attainment	0.01	[−0.03, 0.03]	.991	NA	NA	NA	NA	NA	NA
Time (T3) × attainment	0.01	[−0.03, 0.03]	.782	NA	NA	NA	NA	NA	NA
Time-varying factors									
School climate (teacher)	−1.23	[−2.18, −0.29]	**.011**∗	−0.59	[−1.06, −0.11]	**.015**∗	0.27	[−0.45, 0.99]	.468
Time (T2) × climate	0.60	[−0.25, 1.46]	.166	0.07	[−0.36, 0.51]	.745	−0.45	[−1.14, 0.25]	.212
Time (T3) × climate	0.58	[−0.33, 1.48]	.211	0.39	[−0.06, 0.83]	.091	−0.43	[−1.16, 0.29]	.244
Student-rated school climate									
Student level^a^	−4.25	[−4.48, −4.01]	**< .001**∗	−2.46	[−2.57, −2.35]	**< .001**∗	3.88	[3.70, 4.05]	**< .001**∗
School level	−4.28	[−5.81, −2.75]	**< .001**∗	−2.36	[−3.08, −1.63]	**< .001**∗	4.28	[3.17, 5.38]	**< .001**∗

Note: The baseline assessment (T0) is used as the reference time point except for student-rated school climate, where data were collected only at T1, T2, and T3. Hence, model 2 includes only data collected at T1, T2, and T3, and T1 was used as the reference time point in model 2. Students are nested within schools. NA indicates that associations were not tested because they were nonsignificant in univariable analyses. Given the small numbers observed in our sample and to facilitate data analyses, we coded ethnicity as White and other ethnic groups (including Arab, Asian, Black/African/Caribbean, mixed ethnic groups, other ethnic groups). Boldface type indicates significant values. CES-D = Center for Epidemiological Studies Depression Scale; RI = requires improvement; SDQ = Strengths and Difficulties Questionnaire; T0 = baseline (0 years), T1 = preintervention (1 year), T2 = postintervention (1.5 years), T3 = follow-up (2 years); WEMWBS = Warwick-Edinburgh Mental Well-Being Scale.

a Cluster (school-level) centered.

b Reference: school quality = outstanding.

∗ *p* < .05 (after adjustment for multiple comparisons; Benjamini-Hochberg correction).

For demographic factors, female students had a higher risk of depression score (regression coefficient B = 2.20, 95% CI [1.64, 2.76], *p* < .001) and lower well-being (B = −1.75, 95% CI [−2.22, −1.28], *p* < .001) at baseline. Significant changes in coefficients show that this association strengthened for all outcomes at subsequent assessments. Furthermore, students’ ethnicity (White) was associated with higher social-emotional-behavioral difficulties at baseline (B = 0.80, 95% CI [0.45, 1.15], *p* < .001), which was a stable association across time, present for girls but not boys. Older age was associated with a higher risk of depression (B = 0.53, 95% CI [0.16, 0.90], *p* = .005) and social-emotional-behavioral difficulties (B = 0.41, 95% CI [0.14, 0.67], *p* = .002) and lower well-being (B = −0.87, 95% CI [−1.25, −0.50], *p* < .001) at baseline, which was a stable association over a 2-year period for depression and weakened over time for social-emotional-behavioral difficulties and well-being, particularly for girls. Age was not associated with adolescents’ mental health when considering a 1-year period (model 2).

For school context, students in schools in rural (vs urban) areas had a lower social-emotional-behavioral difficulties score (B = −0.95, 95% CI [−1.66, −0.23], *p* = .009) and higher well-being (B = 1.09, 95% CI [0.28, 1.89], *p* = .008) at baseline. This association was stable across time and present in boys, but not girls. Area-level deprivation was not significantly associated with adolescents’ mental health.

For school community, a higher percentage of White (vs other) students within a school was associated with lower well-being (B = −0.03, 95% CI [−0.04, −0.01], *p* < .001) at baseline. This association was stable across time and present in the whole sample. The mean age of students within a school, the percentage of students eligible for free school meals, and the percentage of students receiving support for special educational needs or disabilities were not significantly associated with adolescents’ mental health at baseline, while minor changes in the relationship were observed at subsequent assessments.

For school operational features, student-rated school climate, analyzed as a time-varying factor at both the student and the school level, was strongly associated with adolescents’ mental health. Better student-rated school climate was associated with a lower risk of depression (student: B = −4.25, 95% CI [−4.48, −4.01], *p* < .001; school: B = −4.28, 95% CI [−5.81, −2.75], *p* < .001), fewer social-emotional-behavioral difficulties (student: B = −2.46, 95% CI [−2.57, −2.35], *p* < .001; school: B = −2.36, 95% CI [−3.08, −1.63], *p* < .001), and higher well-being (student: B = 3.88, 95% CI [3.70, 4.05], *p* < .001; school: B = 4.28, 95% CI [3.17, 5.38], *p* < .001), which was a stable relationship over a 1-year period (T1-T3). These associations were present in girls and boys. Furthermore, better teacher-rated school climate, measured at the school level, was associated with a lower risk of depression score (B = −1.23, 95% CI [−2.18, −0.29], *p* = .011) and lower social-emotional-behavioral difficulties score (B = −0.59, 95% CI [−1.06, −0.11], *p* = .015), which was a stable relation over a 1-year period (T1-T3). However, no effects for teacher-rated school climate were revealed when considering a 2-year period (model 1). Additional small associations were found in crude (unadjusted) models, but were not significant at the 5% level in fully adjusted models (Tables S6-S8).

## Discussion

We explored students’ mental health and well-being during early adolescence and their relationship to individual and school characteristics (context, community, and operational features). Our study suggests 3 key messages: there are significant levels of mental health problems among adolescents, which worsen from ages 11 to 16, particularly in girls; schools account for a small but statistically significant amount of the variation in students’ mental health and well-being; and school climate (particularly as perceived and reported by the students) is the single most important factor associated with these outcomes.

We observed that as children move into mid-adolescence as many as one-third report notable mental health problems. The finding that differences in adolescents’ mental health narrowed over time could be explained by youth who started with worse mental health experiencing improvement, while youth who started with better mental health experienced decline. This could be due to similar but delayed mental health trajectories in the latter or the effect of different vulnerability factors (eg, age or gender), but may equally relate to regression to the mean. Among older girls, the mean score on our measure of risk for depression was above the clinical cutoff point. Moreover, older age, especially among adolescent girls, was significantly associated with worse mental health over time. The rising incidence of mental health problems in the second decade of life, and that the deterioration is particularly marked in girls, is well established.^25^ However, recent evidence suggests that the mental health of young people, especially emotional difficulties in adolescent girls and young women, has deteriorated, which might be due to earlier pubertal timing, maladaptive coping styles (rumination), or a heightened sensitivity to family and peer relationship problems in girls than in boys.^26^ While these findings are significant on the population level (see Table S1, available online), it is important to note that not all adolescents experience mental health symptoms of clinical severity, which underscores the need to consider factors that might protect against the development of mental health difficulties in some adolescents.

Consistent with a previous scoping review and conceptual model,^3^ we found that schools accounted for a small but statistically significant proportion of the variation in adolescents’ mental health. Other factors (eg, schools in urban areas and White ethnicity at the student and school level) were associated with worse mental health in early adolescence but are nonmodifiable. Yet, as regression coefficients were smaller and confidence limits were closer to zero, these factors might be less important in adolescents’ mental health compared with school climate estimates.

As other key modifiable factors are outside the school setting (eg, home connectedness^9^), the role of schools in mental health promotion is important but only part of a wider strategy that should involve all agencies working with children, parents/carers, and families. The green paper “Transforming Children and Young People’s Mental Health Provision”^27^ encourages schools and colleges to promote mental health by identifying a senior mental health lead and building links with mental health support teams. Early evaluation suggests that schools welcome such additional expertise and capacity to support the mental health of their students.^28^ The finding that differences between schools are small suggests that existing school-based approaches target students’ mental health to a similar extent. While similar findings were reported in cross-sectional studies,^6^^,^^10^ the present study applies a theory-based approach to studying a broad range of school-level characteristics and suggests that students in early adolescence with positive views on the school climate had fewer mental health problems and better well-being over 1 year (T1-T3). However, these associations are likely complex and bidirectional (eg, worse mental health being associated with the perception of a worse school climate) and should be further investigated in future experimental studies.

Our findings suggest that schools could enhance the mental health of young people through creating a school climate that students view as positive, including positive peer relationships, caring and respectful adults, and effective school leadership and involvement. This suggestion aligns with the key aims of the Safe and Supportive Schools Project in the United States, which aims to promote school and home connectedness to reduce students’ risk of (mental) health and behavioral problems.^9^ School climate has consistently been associated with a range of important outcomes, including better attendance, less substance use, and better academic performance.^8^ A recent review suggests that establishing peer networks that foster belonging and prosocial behavior could contribute positively to both school climate and student mental health and well-being.^29^

Study strengths include a large, representative sample of UK secondary schools and students, with low percentages of missing data (<2% for our outcomes). The focus on validated measures, appropriate rigorous analyses (multilevel models of repeated measures), and separate mental health outcomes to reveal potential distinct relationships are additional strengths of this study.

The study also has limitations. First, although participating schools were broadly representative of schools in the United Kingdom, the exclusion of schools with inadequate or no SEL strategy limited school variability. Future research should explore how different levels of SEL provision and school quality may influence students’ mental health by oversampling from uncommon school types, including poorly functioning schools. Second, the time window for student- and school-level characteristics to impact students’ mental health over time was narrow, particularly for student ratings of the school climate, which were available only from T1 and hence covered a period of only 1 year. Longer time windows might be needed for school-level effects to emerge. Relations might also change across time and be different for different age groups, which is a question that awaits future research. Nevertheless, the strong prospective associations of student ratings of the school climate with students’ mental health over time suggest that these exploratory findings might persist if longer time periods are considered. Only a subset of teachers and students within each school (ie, participants in the trial) completed school climate ratings,^11^ and there was considerable drop out, particularly of teachers providing school climate ratings over time. Hence, findings might not generalize to the teachers and students who dropped out or nontrial participants, such as students in other year groups not selected for trial participation. Nevertheless, all the reported effects were also identified at the student level, which increases our confidence in the finding that school climate, particularly as perceived by students, is associated with students’ mental health over time. Third, the relationship between school characteristics and students’ mental health is likely complex and bidirectional, meaning that temporal precedence in identified associations needs to be explored further. Fourth, the usual caveats apply regarding the generalizability of the findings to students who did not participate in this study and to schools outside the United Kingdom. However, the fact that students were representative of the wider population and that similar proportions of outcome variation at the school level have been reported in other countries^30^ increases confidence in our findings and their generalizability. Fifth, students retained in this study were more likely to be female and to report lower levels of risk of depression and social-emotional-behavioral difficulties and higher well-being at baseline, which means that caution is warranted when findings are translated to boys or students identifying as other gender identities and for students with worse mental health at baseline. Nevertheless, finding significant adverse effects for girls suggests that we can be more confident in these findings, as girls were more likely to be retained in this study. Furthermore, finding significant effects in students with better mental health at baseline underscores the potential protective role of school climate in students’ mental health over time. Sixth, although the overall trial revealed no differences between school-based mindfulness training and teaching as usual in adolescents’ mental health over time, there remains the potential of unmeasured group differences. Yet, by controlling for trial arm status in the statistical analyses, we minimized this risk, leading to increased confidence in the generalizability of our findings. Finally, there are many variables that impact schools and mental health of young people that were outside the scope of our study, such as nonconforming gender identity,^9^ family-level factors (eg, home connectedness, parental mental health and education),^5^^,^^9^ other school-level factors (eg, percentage of suspensions or bullying/violence),^30^ and the complex interplay of broader influences of inequalities such as poverty or ethnicity.^4^ Future research may explore the relationship between distinct subdimensions of school climate and students’ mental health to obtain a better understanding of the dimensions that are most important, which was beyond the scope of this study.

In summary, our findings are consistent with recent evidence^26^ and suggest worrying levels of poor mental health among UK adolescents, especially girls. Schools account for a small but statistically significant proportion of the variation in mental health of adolescents over time. Using multilevel, multivariable models, we explored associations between a range of theory-driven student- and school-level factors (including school context, community, and operational features) and separate mental health outcomes over time in a representative sample of UK adolescents, entering a higher-risk period of mental health problems. We found a stable role for school climate in students’ mental health in early adolescence in repeated cross-sectional analyses over 1 year. This finding suggests that school climate may play an important role in students’ mental health beyond the influence of other school contextual, community, or operational factors, highlighted in comprehensive frameworks of school-based mental health,^10^ which has important implications for policy and system interventions that focus on enhancing school climate.
