# Supplementary material for: Student- and School-Level Factors Associated With Mental Health and Well-Being in Early Adolescence
Source: J Am Acad Child Adolesc Psychiatry. 2024 Feb;63(2):266–82. doi: 10.1016/j.jaac.2023.10.004 (PMC10935542; doi:10.1016/j.jaac.2023.10.004)
Supplement: Supplemental Material [file mmc1.pdf]

Supplementary Materials

Student- and school-level factors associated with mental health and well-being in early adolescence

Summary of Figures

Figure S1 Adaptation of a Theoretical Model on the Influence of School-Level Factors on Students' Mental Health, as Proposed in Ford et al., 2021[1] 3
Figure S2 Adolescents' Mental Health Growth Curves Overall and by Baseline Mental Health Status 16

Summary of Additional Supplementary Materials

Supplement 1 Eligibility and Representativeness of Schools and Students 4
Supplement 2 Further Information on the Subscales of the Alaska School Climate and Connectedness Survey (SCCS) and Associations with Adolescents' Mental Health 6
Supplement 3 Detailed Information on our Statistical Approach 14

Summary of Tables

Table S1 Differences in Adolescents' Mental Health Between both Trial Arms and over Time 5
Table S2 Key R Packages Used for the Statistical Analyses 13
Table S3 Key Student Baseline Characteristics by 2-Year Follow-Up Status (*N*=8,376) 15
Table S4 Multivariable Analyses (Adjusted for Cohort, Allocation, and Multiple Comparisons) of Repeated Associations for Girls based on the Three-Level Random Intercept Model 19
Table S5. Multivariable Analyses (Adjusted for Cohort, Allocation, and Multiple Comparisons) of Repeated Associations for Boys based on the Three-Level Random Intercept Model 21
Table S6 Univariable Analyses (Unadjusted) of Repeated Associations based on the Three-Level Random Intercept Model 22
Table S7 Univariable Analyses (Unadjusted) of Repeated Associations for Girls based on the Three-Level Random Intercept Model 25
Table S8 Univariable Analyses (Unadjusted) of Repeated Associations for Boys based on the Three-Level Random Intercept Model 27

Figure S1 Adaptation of a Theoretical Model on the Influence of School-Level Factors on Students' Mental Health, as Proposed in Ford et al., 2021[1]

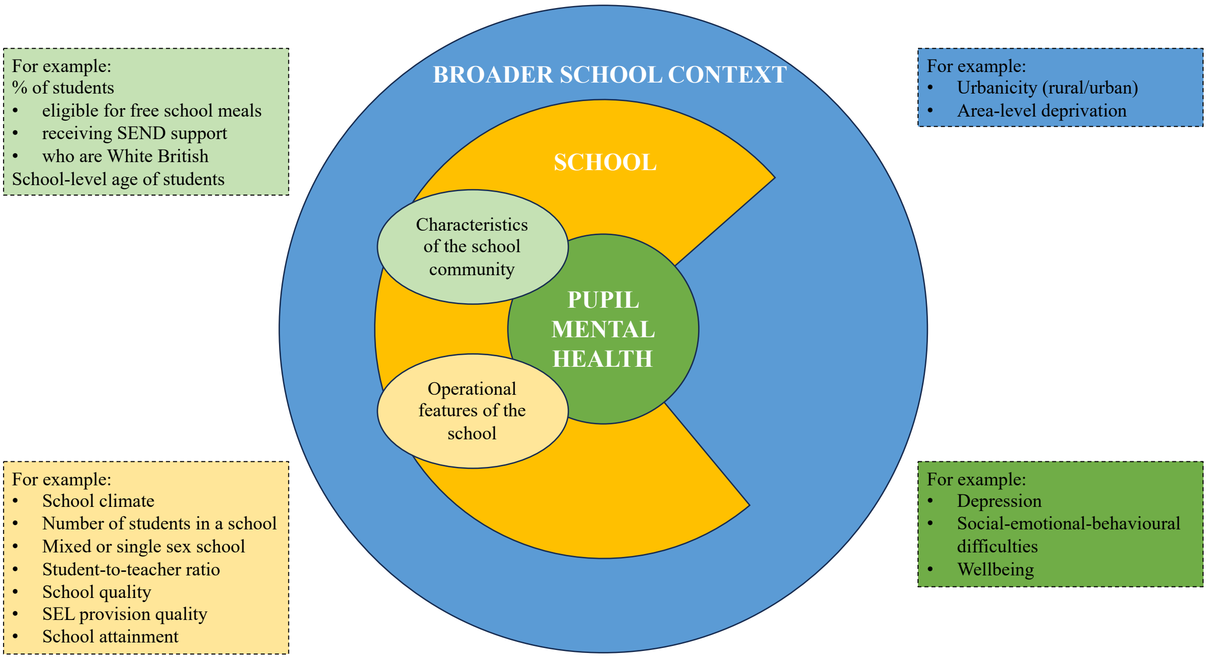

Note: SEL = social and emotional learning; SEND = special educational needs and disability.

Supplement 1 Eligibility and Representativeness of Schools and Students

Eligibility

All students were eligible if they understood English, and all mainstream United Kingdom [UK] secondary schools, including private schools, were eligible if they delivered social-emotional learning [SEL], had not been rated as 'inadequate' in their latest official school inspection (to ensure trial implementation) and had a permanent headteacher.

Representativeness of schools and students

**Schools**	**Target** [a,b]	**Baseline**
**Country**, *n* (%)		
England	68 (80)	75 (88)
Wales	5 (6)	3 (4)
Scotland	8 (9)	3 (4)
Northern Ireland	4 (5)	4 (5)
**School Sex**, *n* (%)		
Single sex	9 (11)	12 (14)
Mixed	76 (89)	73 (86)
**Deprivation**, *n* (%) (% of students eligible for free-school meals in the last 6 years)		
Above average [c]	21 (25)	29 (34)
Below average [c]	64 (75)	56 (66)
**School quality**, *n* (%) [d]		
Outstanding/Excellent/Very good	20 (24)	17 (20)
Good/Satisfactory	47 (55)	46 (54)
Requires improvement/Adequate	14 (16)	11 (13)
Not yet rated/NA/UNK	4 (5)	11 (13)
**School type**, *n* (%)		
Non-selective	74 (87)	74 (87)
Selective	4 (5)	8 (9)
Independent	7 (8)	3 (4)
**School size**, *n* (%)		
Small (<1000) [e]	45 (53)	39 (46)
Large (≥1000) [e]	40 (47)	46 (54)
**Students**	**Normative**	**Baseline**
Gender, female, *n* (%)	(49.0)	4509 (55)
Ethnicity, White, *n* (%)	(77.3)	6202 (76)
Risk for depression, mean (SD)	13.9 (9.7) [f]	13.5 (9.9)
Social-emotional-behavioural difficulties, mean (SD)	10.3 (5.2) [g]	11.8 (6.5)
Well-being, mean (SD)	48.8 (6.8) [h]	49.7 (9.7)

[a] Kuyken et al., (2017).[2]
[b] Kuyken et al., (2022).[3]
[c] National average: 13.2% (Department for Education, 2016), last 6 years: 29.4% (Department of Education, 2020)
[d] Each country within the UK has its own school's inspectorate, each with a different quality rating system. As only 12 schools were evaluated by a different inspectorate than Ofsted (England's state-funded schools inspectorate), the school quality ratings for these 12 schools were mapped onto the Ofsted rating system to allow for an analyses of school quality ratings across schools. Two researchers independently reviewed the published, inspectorate reports for these 12 schools at the time the schools entered the project, to assign quality ratings from 1-4, in keeping with the Ofsted quality ratings (1=highest quality to 4=lowest quality; inter-rater reliability: 91%).
[e] School size: cut off = 1,000.
[f] Briere et al., (2013).[4]
[g] Strengths and Difficulties Questionnaire (SDQ) norms.[5]
[h] Clarke et al. (2011).[6]

Source data

We used publicly available data (e.g., Department of Education, 2020; https://gov.uk), collected closest to the year in which students' provided baseline (T0) data.
Free school meals - % eligible in 2016: 13.2%
https://assets.publishing.service.gov.uk/government/uploads/system/uploads/attachment_data/file/552342/SFR20_2016_Main_Text.pdf
Free school meals - % eligible in last 6 years = 29.4%
https://www.gov.uk/government/statistics/schools-pupils-and-their-characteristics-january-2020
OFSTED rating: https://reports.ofsted.gov.uk/
Further information on school size and type was sourced from: https://www.compare-school-performance.service.gov.uk/

Table S1 Differences in Adolescents' Mental Health Between both Trial Arms and over Time

		T0		T1			T2			T3		
		n	mean (SD)	*n*	mean (SD)	*d* [95% CI]	*n*	mean (SD)	*d* [95% CI]	*n*	mean (SD)	*d* [95% CI]
CES-D	SBMT	n/a	n/a	4,073	15.6 (11.2)		3,768	16.9 (11.8)		3,672	17.1 (11.9)	
	TAU	n/a	n/a	3,981	15.6 (10.9)		3,793	16.4 (11.6)		3,566	16.6 (11.9)	
	Total	8,370	13.5 (9.9)	8,054	15.6 (11.1)	0.20 [0.23,0.17]	7,561	16.6 (11.7)	0.29 [0.33,0.26]	7,238	16.9 (11.9)	0.31 [0.35,0.28]
SDQ	SBMT	n/a	n/a	4,065	12.5 (6.7)		3,752	13.4 (6.9)		3,664	13.2 (6.8)	
	TAU	n/a	n/a	3,977	12.3 (6.5)		3,790	13.1 (6.9)		3,561	12.9 (6.8)	
	Total	8,252	11.8 (6.5)	8,042	12.4 (6.6)	0.09 [0.13,0.06]	7,542	13.3 (6.9)	0.22 [0.25,0.19]	7,225	13.1 (6.8)	0.20 [0.23,0.16]
WEMWBS	SBMT	n/a	n/a	4,072	49.2 (9.2)		3,775	47.8 (9.6)		3,678	47.6 (9.8)	
	TAU	n/a	n/a	3,986	49.0 (9.0)		3,797	48.1 (9.4)		3,566	47.6 (9.8)	
	Total	8,333	49.7 (9.7)	8,058	49.1 (9.1)	-0.06 [-0.03,-0.09]	7,572	47.9 (9.5)	-0.18 [-0.15,-0.21]	7,244	47.6 (9.8)	-0.21 [-0.18,-0.25]

Note: Cohen's *d* compares the baseline (T0) means and SDs in the whole sample to the respective values at T1, T2, and T3. CES-D = Center for Epidemiologic Studies for Depression Scale. n/a = Participants were assigned to the intervention or control group after the T0 assessment and prior to the T1 assessment. SBMT = school-based mindfulness training. SDQ = Strengths and Difficulties Questionnaire. TAU = treatment as usual. WEMWBS = Warwick-Edinburgh Mental Well-Being Scale.

Supplement 2 Further Information on the Subscales of the Alaska School Climate and Connectedness Survey (SCCS) and Associations with Adolescents' Mental Health

School climate was defined as teachers' and students' experiences in a school, including feeling safe, connected, and welcomed. It includes connections, partnerships, and conditions for learning. The Alaska School Climate and Connectedness Survey [(SCCS]) was used to assess teacher-rated (subscales: School Leadership and Involvement; Staff Attitudes; and Respectful Climate) and student-rated (subscales: School leadership and Involvement; Respectful Climate; Peer Climate; and Caring Adults) school climate, with higher total scores (range: 1-5) representing a better school climate. We chose school climate subscales that were hypothesised to be most relevant and sensitive to change following the mindfulness intervention, without losing important aspects of the original construct. Using confirmatory factor analysis (reported below), we confirmed that this reduced measure remains an appropriate measure of the underlying school climate construct.

Teachers

School Leadership and Involvement

Items				
I trust the principal will keep his or her word.				
The principal looks out for the personal welfare of school staff members.				
The principal and other leaders in this school make good decisions.				
I am satisfied with my involvement in decision making at this school.				
The work rules at this school are fair.				
School staff members have a lot of informal opportunities to influence what happens here.				
When students break the rules they are treated fairly.				
At school, decisions are made based on what is best for students.				
Response range: 1-5, where 1 indicates a low level of school leadership and 5 indicates a high level of school leadership.				
Internal consistencies (Original[a]: α = .93)	MYRIAD sample			
	T0: α = .88	T1: α = .90	T2: α = .92	T3: α = .91

Staff Attitudes

Items				
The teachers at this school are good at their jobs.				
Teachers here set high standards for themselves.				
Teachers here are nice people.				
In this school, staff members have a "can do" attitude.				
Teachers and staff believe that *all* students can do good work.				
Response range: 1-5, where 1 indicates more negative staff attitudes and 5 indicates a more positive staff attitudes.				
Internal consistencies (Original[a]: α = .86)	MYRIAD sample			
	T0: α = .81	T1: α = .82	T2: α = .83	T3: α = .83

Respectful Climate

Items				
Students in this school treat each other with respect.				
Students in this school help each other, even if they are not friends.				
Teachers and students treat each other with respect in this school.				
The students in this school don't really care about each other. (reverse scored)				
At this school, students and teachers get along really well.				
Response range: 1-5, where 1 indicates a low level of respect and 5 indicates a high level of respect.				
Internal consistencies (Original[a]: α = .86)	MYRIAD sample			
	T0: α = .84	T1: α = .85	T2: α = .86	T3: α = .84

The subscales 'Student involvement', 'School safety', and 'Parental and community involvement' were omitted in the MYRIAD trial.

[a] The Association of Alaska School Boards, 2010. *2010 School Climate and Connectedness Survey: Statewide Report*. American Institute for Research.

Factor models

Timepoint	*n*	*N*	Robust Chi-square	*df*	*p*-value	CFI	TLI	RMSEA [90% CI]	SRMR
T0	678	679	550.9	132	< 0.001	0.911	0.897	0.068 [0.063; 0.074]	0.060
T1	491	679	438.6	132	< 0.001	0.919	0.906	0.069 [0.062; 0.076]	0.055
T2	394	679	368.4	132	< 0.001	0.931	0.920	0.067 [0.060;0.075]	0.054
T3	382	679	388.5	132	< 0.001	0.919	0.906	0.071 [0.063; 0.079]	0.058

Note: Models were estimated via robust maximum likelihood estimation for all trial teachers with available data at the T0 assessment (the first time they were asked to complete the school climate measure). We accounted for missing data using full-information maximum likelihood (FIML). Comparative Fit Index = CFI (values greater than 0.90 indicate good fit); *N* = sample size following FIML estimation, which we used to obtain our model estimates; *n* = sample size without missing data; Root Mean Square Error of Approximation = RMSEA (≤ 0.05 (close-fit), .05 to .08 (reasonable approximate fit), ≥0.10 (poor-fit)); Standardized Root Mean Square Residual = SRMR (<0.10 (acceptable fit)); Tucker-Lewis Index = TLI (values greater than 0.90 indicate good fit).

Factor model showing the standardised estimates at each timepoint

T0:

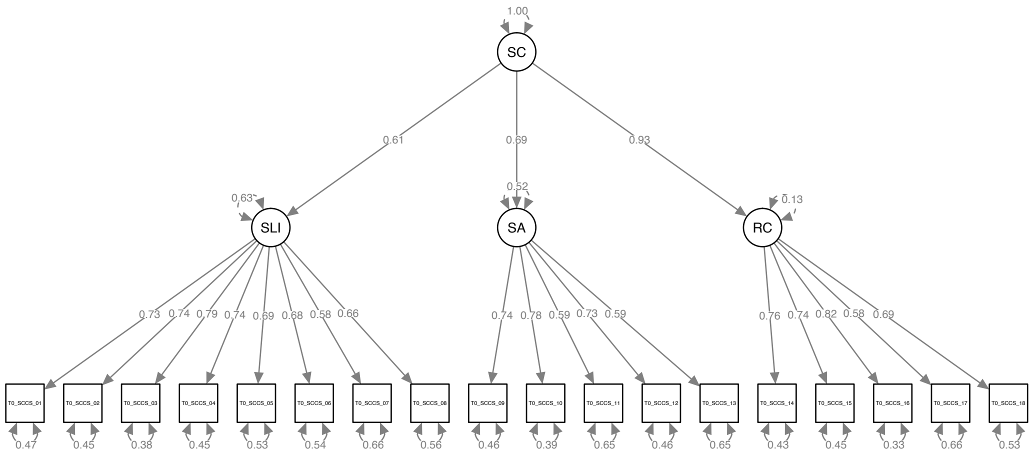

T1:

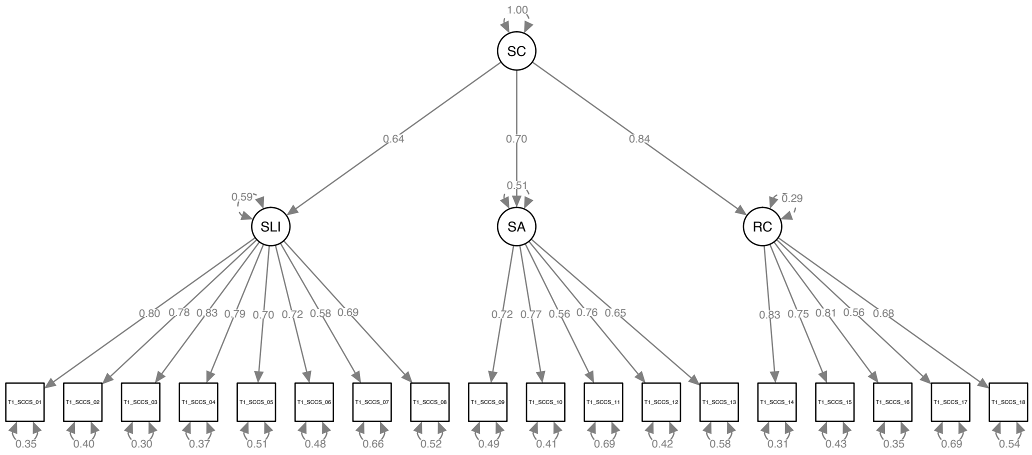

T2:

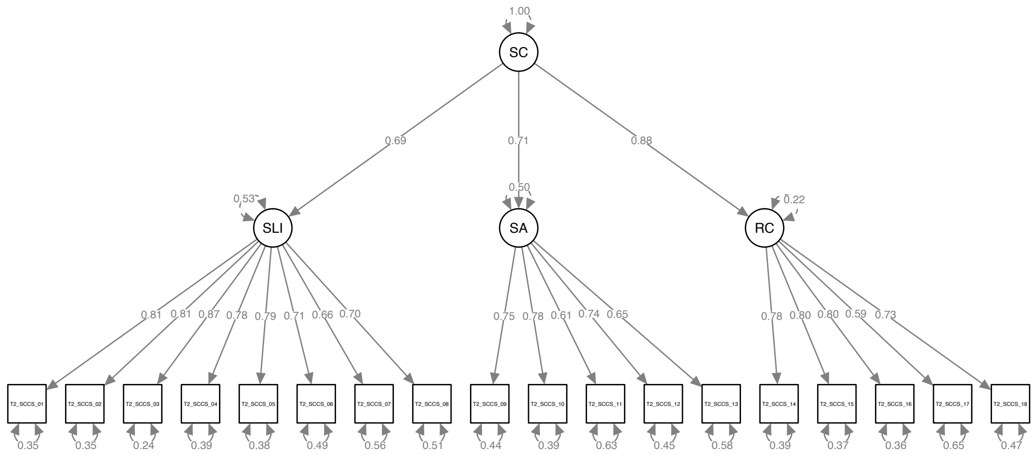

T3:

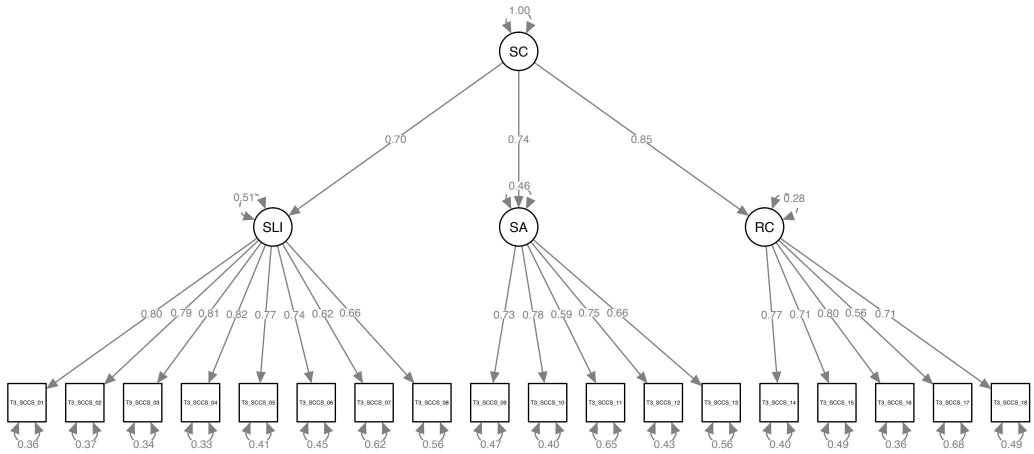

	**R-Square**			
Item	T0	T1	T2	T3
1. I trust the principal will keep his or her word.	0.528	0.646	0.652	0.639
2. The principal looks out for the personal welfare of school staff members.	0.545	0.604	0.654	0.630
3. The principal and other leaders in this school make good decisions.	0.620	0.697	0.760	0.662
4. I am satisfied with my involvement in decision making at this school.	0.549	0.627	0.607	0.672
5. The work rules at this school are fair.	0.471	0.489	0.623	0.588
6. School staff members have a lot of informal opportunities to influence what happens here.	0.463	0.516	0.509	0.549
7. When students break the rules they are treated fairly.	0.336	0.337	0.436	0.382
8. At school, decisions are made based on what is best for students.	0.441	0.476	0.486	0.436
School Leadership and Involvement (SLI)	0.370	0.407	0.471	0.488
9. The teachers at this school are good at their jobs.	0.543	0.512	0.565	0.533
10. Teachers here set high standards for themselves.	0.606	0.594	0.609	0.602
11. Teachers here are nice people.	0.349	0.312	0.368	0.352
12. In this school, staff members have a "can do" attitude.	0.539	0.585	0.547	0.566
13. Teachers and staff believe that *all* students can do good work.	0.353	0.423	0.422	0.440
Staff Attitudes (SA)	0.476	0.486	0.501	0.545
14. Students in this school treat each other with respect.	0.570	0.693	0.609	0.598
15. Students in this school help each other, even if they are not friends.	0.549	0.568	0.635	0.508
16. Teachers and students treat each other with respect in this school.	0.667	0.653	0.642	0.636
17. The students in this school don't really care about each other. (reverse scored)	0.340	0.314	0.352	0.316
18. At this school, students and teachers get along really well.	0.471	0.457	0.528	0.507
Respectful Climate (RC)	0.869	0.713	0.782	0.720

Students

School Leadership and Involvement

Items			
At school, decisions are made based on what is best for students.			
The principal and other leaders in this school make good decisions.			
In my school, students are given a chance to help make decisions.			
Students are involved in helping to solve school problems.			
The principal asks students about their ideas.			
Response range: 1-5, where 1 indicates a low level of school leadership and student involvement and 5 indicates a high level of school leadership and student involvement.			
Internal consistencies (Original[a]: α = .80)	MYRIAD sample		
	T1: α = .84	T2: α = .86	T3: α = .88

Respectful Climate

Items			
Teachers here are nice people.			
My teachers treat me with respect.			
When students break rules, they are treated fairly.			
My teachers are fair.			
Our school rules are fair.			
It pays to follow the rules at my school.			
Response range: 1-5, where 1 indicates a lack of a respectful climate and 5 indicates a highly respectful climate for students.			
Internal consistencies (Original[a]: α = .85)	MYRIAD sample		
	T1: α = .87	T2: α = .87	T3: α = .88

Peer Climate

Items			
Students in this school help each other, even if they are not friends.			
Students here treat me with respect.			
When students see another student being picked on they try to stop it.			
Students at this school are often teased or picked on. (reversed scored)			
Most students in this school like to put others down. (reverse scored)			
Response range: 1-5, where 1 indicates a more negative peer climate and student involvement and 5 indicates a more positive peer climate at the school.			
Internal consistencies (Original[a]: α = .72)	MYRIAD sample		
	T1: α = .77	T2: α = .78	T3: α = .77

Caring Adults

Items			
There is at least one adult at this school whom I feel comfortable talking to about things that are bothering me.			
At school, there is a teacher or some adult who will miss me when I'm absent.			
There are a lot of chances for students in my school to talk to teachers one on one.			
I can name at least five adults who really care about me.			
Other adults at school besides my teachers know my name.			
Response range: 1-5, where 1 indicates a low number of adult caring relationships and 5 indicates a high number of adult caring relationships.			
Internal consistencies (Original[a]: α = .69)	MYRIAD sample		
	T1: α = .73	T2: α = .75	T3: α = .77

The subscales 'High expectations', 'School safety', and 'Parental and community involvement' were omitted in the MYRIAD trial.

[a] The Association of Alaska School Boards, 2010. *2010 School Climate and Connectedness Survey: Statewide Report*. American Institute for Research.

Factor models

Timepoint	*n*	*N*	Robust Chi-square	*df*	*p*-value	CFI	TLI	RMSEA [90% CI]	SRMR
T1	7805	8072	5267.8	185	< 0.001	0.904	0.891	0.059 [0.058; 0.061]	0.046
T2	7168	8072	6133.9	185	< 0.001	0.886	0.871	0.067 [0.066; 0.068]	0.053
T3	6922	8072	6787.8	185	< 0.001	0.878	0.861	0.072 [0.071; 0.073]	0.058

Note: Models were estimated via robust maximum likelihood estimation for all students with available data at the T1 assessment (the first time they were asked to complete the school climate measure). We accounted for missing data using full-information maximum likelihood (FIML). Comparative Fit Index = CFI (values greater than 0.90 indicate good fit); *N* = sample size following FIML estimation, which we used to obtain our model estimates; *n* = sample size without missing data; Root Mean Square Error of Approximation = RMSEA (≤ 0.05 (close-fit), .05 to .08 (reasonable approximate fit), ≥0.10 (poor-fit)); Standardized Root Mean Square Residual = SRMR (<0.10 (acceptable fit)); Tucker-Lewis Index = TLI (values greater than 0.90 indicate good fit).

Factor model showing the standardised estimates at each timepoint

T1:

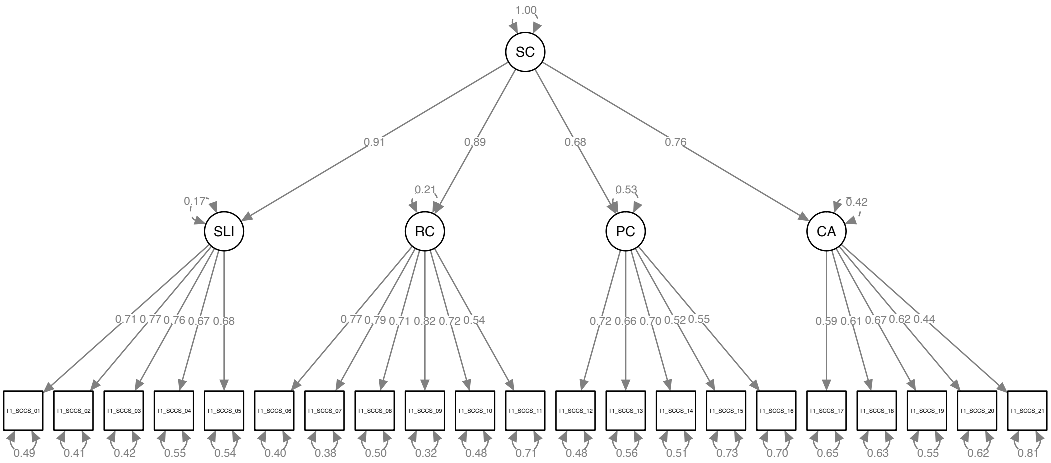

T2:

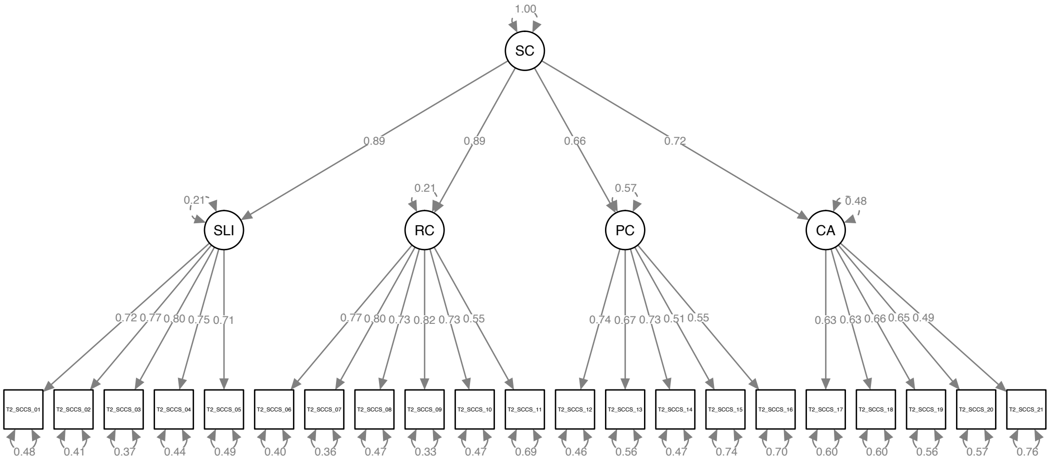

T3:

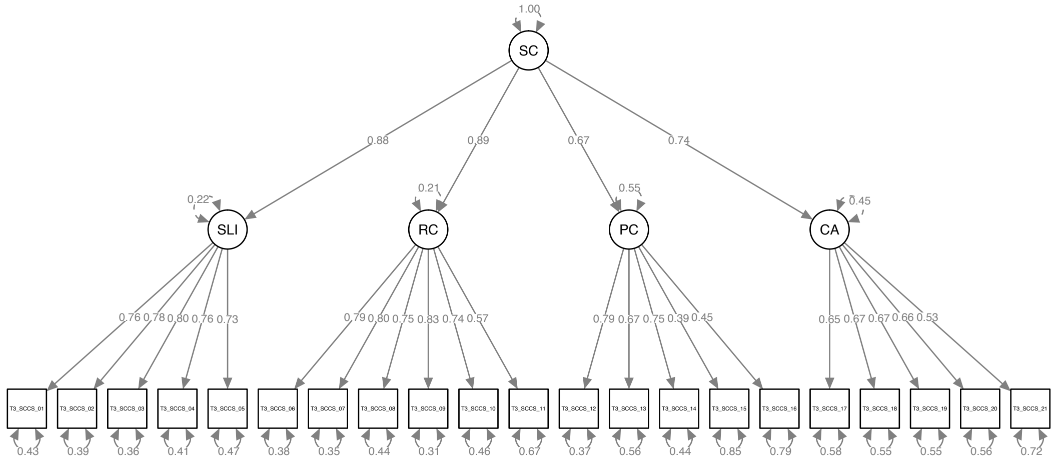

Item	R-Square		
	T1	T2	T3
1. At school, decisions are made based on what is best for students.	0.511	0.523	0.574
2. The principal and other leaders in this school make good decisions.	0.588	0.588	0.614
3. In my school, students are given a chance to help make decisions.	0.576	0.633	0.645
4. Students are involved in helping to solve school problems.	0.450	0.558	0.585
5. The principal asks students about their ideas.	0.458	0.507	0.526
School Leadership and Involvement (SLI)	0.828	0.786	0.781
6. Teachers here are nice people.	0.600	0.598	0.624
7. My teachers treat me with respect.	0.619	0.640	0.648
8. When students break rules, they are treated fairly.	0.501	0.530	0.563
9. My teachers are fair.	0.678	0.675	0.692
10. Our school rules are fair.	0.520	0.530	0.542
11. It pays to follow the rules at my school.	0.289	0.305	0.328
Respectful Climate (RC)	0.793	0.786	0.789
12. Students in this school help each other, even if they are not friends.	0.524	0.543	0.626
13. Students here treat me with respect.	0.437	0.445	0.443
14. When students see another student being picked on they try to stop it.	0.489	0.529	0.563
15. Students at this school are often teased or picked on. (reversed scored)	0.266	0.261	0.149
16. Most students in this school like to put others down. (reverse scored)	0.301	0.300	0.205
Peer Climate (PC)	0.469	0.429	0.454
17. There is at least one adult at this school whom I feel comfortable talking to about things that are bothering me.	0.353	0.399	0.423
18. At school, there is a teacher or some adult who will miss me when I'm absent.	0.369	0.401	0.448
19. There are a lot of chances for students in my school to talk to teachers one on one.	0.449	0.440	0.449
20. I can name at least five adults who really care about me.	0.382	0.428	0.437
21. Other adults at school besides my teachers know my name.	0.190	0.235	0.278
Caring Adults (CA)	0.582	0.525	0.550

Cross-sectional associations between school-climate subscales and adolescents' mental health over time

Correlations at T0

	Risk for depression (CES-D) *r* [95%CI]	Social-emotional-behavioural difficulties (SDQ) *r* [95%CI]	Wellbeing (WEMWBS) *r* [95%CI]
**School-level teacher-ratings of the school climate (SCCS)**			
School Leadership and Involvement	-0.01 [-0.03; 0.01]	-0.03 [-0.06; -0.01]	0.03 [0.01; 0.05]
Staff Attitudes	-0.03 [-0.05; -0.01]	-0.05 [-0.07; -0.03]	0.02 [-0.01; 0.04]
Respectful Climate	-0.02 [-0.04; -0.01]	-0.06 [-0.08; -0.04]	0.02 [0.01; 0.05]

Correlations at T1

	Risk for depression (CES-D) *r* [95%CI]	Social-emotional-behavioural difficulties (SDQ) *r* [95%CI]	Wellbeing (WEMWBS) *r* [95%CI]
**School-level teacher-ratings of the school climate (SCCS)**			
School Leadership and Involvement	0.01 [-0.01; 0.03]	-0.02 [-0.04; -0.01]	0.01 [-0.02; 0.03]
Staff Attitudes	0.00 [-0.02; 0.02]	-0.04 [-0.06; -0.02]	0.00 [-0.03; 0.01]
Respectful Climate	0.00 [-0.03; 0.02]	-0.05 [-0.07; -0.03]	0.02 [-0.01; 0.04]
**School-level student-ratings of the school climate (SCCS)**			
School Leadership and Involvement	-0.07 [-0.09; -0.05]	-0.06 [-0.08; -0.04]	0.08 [0.05; 0.10]
Respectful Climate	-0.08 [-0.10; -0.06]	-0.07 [-0.09; -0.05]	0.07 [0.05; 0.09]
Peer Climate	-0.06 [-0.08; -0.04]	-0.09 [-0.12; -0.07]	0.08 [0.05; 0.10]
Caring Adults	-0.11 [-0.13; -0.09]	-0.06 [-0.08; -0.04]	0.11 [0.09; 0.13]
**Student-level student-ratings of the school climate (SCCS)**			
School Leadership and Involvement	-0.29 [-0.31; -0.27]	-0.34 [-0.35; -0.32]	0.35 [0.33; 0.37]
Respectful Climate	-0.32 [-0.34; -0.30]	-0.38 [-0.40; -0.36]	0.35 [0.33; 0.37]
Peer Climate	-0.43 [-0.45; -0.42]	-0.47 [-0.49; -0.46]	0.42 [0.40; 0.43]
Caring Adults	-0.34 [-0.36; -0.32]	-0.31 [-0.33; -0.29]	0.40 [0.38; 0.42]

Correlations at T2

	Risk for depression (CES-D) *r* [95%CI]	Social-emotional-behavioural difficulties (SDQ) *r* [95%CI]	Wellbeing (WEMWBS) *r* [95%CI]
**School-level teacher-ratings of the school climate (SCCS)**			
School Leadership and Involvement	0.01 [-0.01; 0.03]	-0.01 [-0.04; 0.01]	-0.01 [-0.04; 0.01]
Staff Attitudes	0.01 [-0.01; 0.04]	-0.01 [-0.04; 0.01]	-0.03 [-0.06; -0.01]
Respectful Climate	0.02 [-0.01; 0.04]	-0.02 [-0.05; -0.01]	0.00 [-0.03; 0.02]
**School-level student-ratings of the school climate (SCCS)**			
School Leadership and Involvement	-0.04 [-0.06; -0.02]	-0.05 [-0.08; -0.03]	0.04 [0.02; 0.07]
Respectful Climate	-0.06 [-0.08; -0.04]	-0.07 [-0.09; -0.04]	0.05 [0.03; 0.08]
Peer Climate	-0.04 [-0.06; -0.02]	-0.08 [-0.10; -0.06]	0.05 [0.02; 0.07]
Caring Adults	-0.10 [-0.12; -0.07]	-0.05 [-0.07; -0.03]	0.09 [0.07; 0.12]
**Student-level student-ratings of the school climate (SCCS)**			
School Leadership and Involvement	-0.28 [-0.30; -0.26]	-0.30 [-0.32; -0.28]	0.30 [0.28; 0.32]
Respectful Climate	-0.30 [-0.32; -0.28]	-0.35 [-0.37; -0.33]	0.32 [0.30; 0.34]
Peer Climate	-0.41 [-0.43; -0.40]	-0.45 [-0.46; -0.43]	0.38 [0.36; 0.40]
Caring Adults	-0.33 [-0.35; -0.31]	-0.30 [-0.32; -0.28]	0.38 [0.36; 0.40]

Correlations at T3

	Risk for depression (CES-D) *r* [95%CI]	Social-emotional-behavioural difficulties (SDQ) *r* [95%CI]	Wellbeing (WEMWBS) *r* [95%CI]
**School-level teacher-ratings of the school climate (SCCS)**			
School Leadership and Involvement	-0.02 [-0.04; 0.01]	-0.02 [-0.04; 0.01]	0.02 [-0.01; 0.04]
Staff Attitudes	-0.01 [-0.03; 0.01]	-0.01 [-0.04; 0.01]	0.01 [-0.02; 0.03]
Respectful Climate	0.00 [-0.02; 0.02]	-0.03 [-0.05; -0.01]	0.02 [-0.01; 0.04]
**School-level student-ratings of the school climate (SCCS)**			
School Leadership and Involvement	-0.05 [-0.08; -0.03]	-0.04 [-0.07; -0.02]	0.05 [0.02; 0.07]
Respectful Climate	-0.06 [-0.09; -0.04]	-0.06 [-0.08; -0.03]	0.05 [0.03; 0.07]
Peer Climate	-0.04 [-0.06; -0.02]	-0.07 [-0.09; -0.05]	0.05 [0.02; 0.07]
Caring Adults	-0.10 [-0.12; -0.08]	-0.05 [-0.07; -0.03]	0.10 [0.08; 0.12]
**Student-level student-ratings of the school climate (SCCS)**			
School Leadership and Involvement	-0.27 [-0.29; -0.25]	-0.31 [-0.33; -0.29]	0.27 [0.24; 0.29]
Respectful Climate	-0.30 [-0.33; -0.28]	-0.36 [-0.38; -0.34]	0.30 [0.28; 0.32]
Peer Climate	-0.40 [-0.42; -0.38]	-0.44 [-0.45; -0.42]	0.35 [0.33; 0.37]
Caring Adults	-0.34 [-0.36; -0.32]	-0.32 [-0.34; -0.30]	0.37 [0.35; 0.39]

Note: CES-D = Center for Epidemiologic Studies for Depression Scale; SCCS = School Climate and Connectedness Survey. SDQ = Strengths and Difficulties Questionnaire; WEMWBS = Warwick-Edinburgh Mental Well-Being Scale.

Table S2 Key R Packages Used for the Statistical Analyses

Analysis part	R package (version number)	Reference
Descriptive statistics	dplyr (1.0.8)	Wickham, François, Henry, & Müller (2022)[7]
	Hmisc (4.6-0)	Harrell (2021)[8]
	psych (2.1.9)	Revelle (2021)[9]
	base (3.6.2)	R Core Team (2019)[10]
Data preparation	tidyr (1.2.0)	Wickham & Girlich (2022)[11]
	misty (0.4.3)	Yanagida (2021)[12]
	readspss (0.14)	Garbuszus & Pfaff (2021)[13]
Graphical visualisations	ggplot2 (3.3.5)	Wickham (2016)[14]
	sjPlot (2.8.9)	Lüdecke (2021)[15]
	graphics (3.6.2)	R Core Team (2019)[10]
Multilevel growth analysis	lme4 (1.1-27.1)	Bates, Maechler, Bolker, & Walker (2015)[16]
	car (3.0-12)	Fox, & Weisberg (2019)[17]
	parameters (0.15.0)	Lüdecke, Ben-Shachar, Patil, & Makowski (2020)[18]
Assumptions/ correction for multiple comparisons/ ordinary linear regression analysis	stats (3.6.2)	R Core Team (2019)[10]
	performance (0.8.0)	Lüdecke, Ben-Shachar, Patil, Waggoner, & Makowski, (2021)[19]
Confirmatory factor analysis	lavaan (0.6-9)	Rosseel (2012)[20]
Effect size	effsize (0.8.1)	Torchiano (2020)[21]

Supplement 3 Detailed Information on our Statistical Approach
All statistical analyses were undertaken in R version 3.6.2[10] (the main R packages and version numbers are listed in the supplementary Table S2). We summarised participant characteristics, using means and standard deviations for continuous variables and counts and percentages for categorical variables. Growth plots were used to display individual and mean mental health growth curves for our three co-primary outcomes (depression, social-emotional-behavioural difficulties, and well-being), which were analysed separately throughout this manuscript. We also plotted growth curves (T1-T3) by prior mental health status (baseline T0 levels), using established cut-off scores. We conducted complete-case analyses and explored differences between students retained and lost to follow-up, using the Welch two sample t-test for continuous data and the Pearson's chi-squared test for categorical data.

To estimate the intra-cluster (i.e., school) correlation coefficient (ICCs), we fitted two-level random intercept multilevel linear regression models for each outcome and timepoint, using maximum likelihood estimation (level 1: student-level and level 2: school-level). In a two-level random intercept model, the ICC is the proportion of variation in adolescents' mental health that is between schools: $ICC = \frac{v11}{v11+\sigma2}$.[22] Here, $v22$ is the variance component for the school-level and σ^2 is the residual variance. Bootstrapping (N=100) was used to obtain 95% confidence intervals for the ICCs.

We undertook a series of analyses to examine changes over time in the repeated associations between student- and school-level factors and mental health outcomes (time: categorical: 0 (reference), 1, 1.5, and 2 years). These changes either refer to changes in the cross-sectional associations over time (i.e., for student- and teacher-rated school climate factors, assessed repeatedly at each timepoint (time-varying)) or changes in the longitudinal associations (i.e., for all other student- and school-level factors, assessed only at baseline (time-constant)). For this, we fitted three-level random intercept multilevel linear regression models, using maximum likelihood estimation (level 1: repeated measures over time within students, level 2: student, and level 3: school). All continuous level-2 factors (i.e., student-level age and student-level student-rated school climate) were centred at the cluster (i.e., school) level to obtain regression coefficients that represent the predicted change in the outcome that is associated with within-school increases in the predictors (i.e., differences between students within schools). All analyses were then stratified by gender (self-identified boys and girls). For boys, we excluded the school-level factor "school sex" to avoid the disclosure risks of students identifying as boys in female-only schools. In exceptional cases model convergences problems, due to high tolerance values, were resolved by using a quadratic approximation (optimiser "bobyqa").[23] Where applicable, we used 2-sided contrasts with a significance level of 0.05 and interpreted the 95% confidence intervals.

The analyses of associations between the factors and the outcomes were undertaken as follows. First, we examined one factor at a time. Models with the time-by-factor interaction term, were compared to models with only the respective main effects of time and the factor. If the inclusion of this interaction term provided a better model fit (likelihood ratio test [LRT]: $p < 0.05$), then this was used as evidence that the association between the factor and the outcome changes over time. Thus, regression coefficients for the time-by-factor interaction term reflect changes in the relationship relative to the first assessment (i.e., T1/T2/T3 vs. T0 (reference); T1 is the reference timepoint for student-rated school climate). In the absence of statistically significant interactions with time (LRT: $p > 0.05$), we assumed stability in the relationship and regression coefficients reflect the average relationship across time.

Second, we included statistically significant main or time-by-factor interaction terms in a series of up to two multivariable models, where we adjusted for cohort, trial arm (allocation), and multiple comparisons (Benjamini-Hochberg correction): Model 1 (student-level demographics + school context + school community + operational features, except student-rated school climate) and Model 2 (all factors in model 1 + student-rated school climate). Model 1 includes as repeated measures adolescents' mental health outcomes at T0-T3, whilst Model 2 includes outcomes at T1-T3.

Table S3 Key Student Baseline Characteristics by 2-Year Follow-Up Status (*N*=8,376)

Variables	Students lost to follow-up	Remaining students	Group differences
	**Total (*n* = 1,126)**	**Total (*n* = 7,250)**	***p*-value [f]**
Age, mean (SD)	12.2 (0.6)	12.2 (0.6)	$p = 0.317$
Gender [a]			**$p = 0.012$**
female, *n* (%)	562 (51.1)	3,947 (55.4)	
male, *n* (%)	509 (46.3)	3,038 (42.7)	
other, *n* (%)	7 (0.6)	19 (0.3)	
prefer not to say, *n* (%)	22 (2.0)	116 (1.6)	
Ethnicity – White [b], *n* (%)	824 (75.3)	5,378 (75.8)	$p = 0.739$
Depression (CES-D) [c], mean (SD)	15.5 (10.7)	13.1 (9.7)	**$p < 0.001$**
Social-emotional-behavioural difficulties (SDQ) [d], mean (SD)	13.5 (6.6)	11.5 (6.4)	**$p < 0.001$**
Well-being (WEMWBS) [e], mean (SD)	48.2 (10.2)	49.9 (9.6)	**$p < 0.001$**

Note: Students lost to follow-up were defined as those students with missing data on all 3 co-primary outcomes at follow-up. Remaining students were defined as those students with at least one of the co-primary outcomes at follow-up. Given the small numbers observed in our sample and to facilitate data analyses, we coded 'Ethnicity' as 'White' and 'other ethnic groups' (incl. Arab, Asian, Black/African/Caribbean, mixed ethnic groups, other ethnic groups). CES-D = Center for Epidemiologic Studies for Depression Scale; SDQ = Strengths and Difficulties Questionnaire; WEMWBS =Warwick-Edinburgh Mental Well-Being Scale.

[a] Sample size in lost to follow-up group: 1,100. Sample size those remaining: 7,120.

[b] Sample size in lost to follow-up group: 1,095. Sample size those remaining: 7,098.

[c] Sample size those remaining: 7,244.

[d] Sample size in lost to follow-up group: 1,104. Sample size those remaining: 7,148.

[e] Sample size in lost to follow-up group: 1,120. Sample size those remaining: 7,213.

[f] For continuous factors this was derived from the Welch two sample t-test, for categorical data this was derived from the Pearson's chi-squared test. Significant group differences are highlighted in bold.

Figure S2 Adolescents' Mental Health Growth Curves Overall and by Baseline Mental Health Status

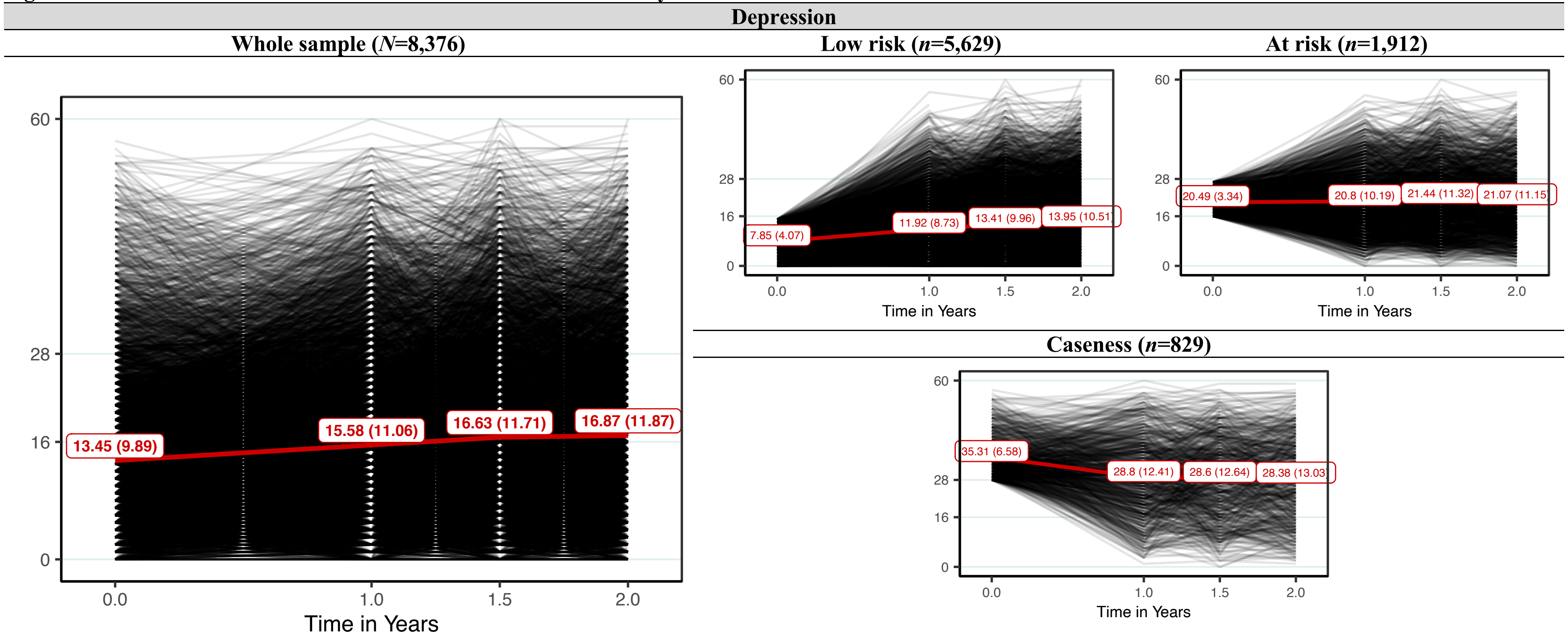

Note: Cut-off scores are based on the official scoring guidelines. Depression: low (0–15); at risk of depression (16–27); caseness (28–60).[24,25]

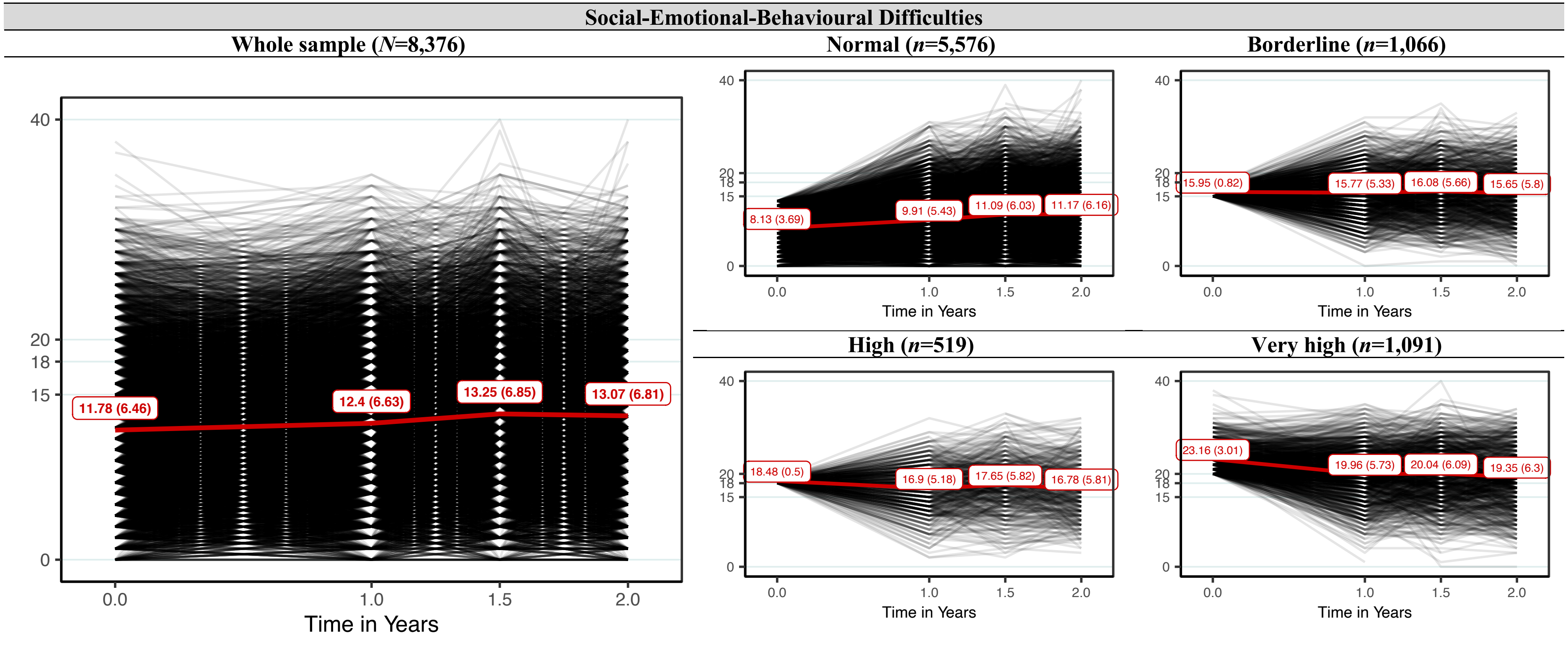

Note: Cut-off scores are based on the official scoring guidelines. Social-emotional-behavioural difficulties: normal (0–14); borderline (15–17); high (18–19); very high (20–40).[26]

Well-being

Whole sample (*N*=8,376)

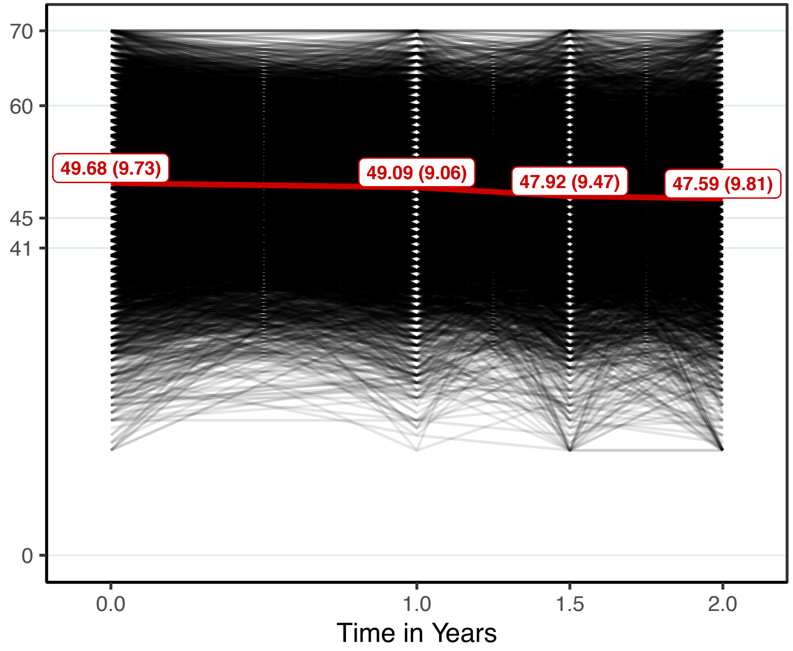

Probable Mental Health Difficulties (*n*=1,445)

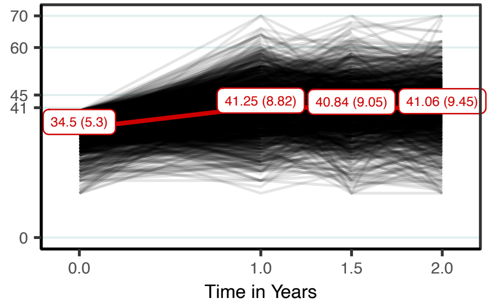

Possible Mental health Difficulties (*n*=963)

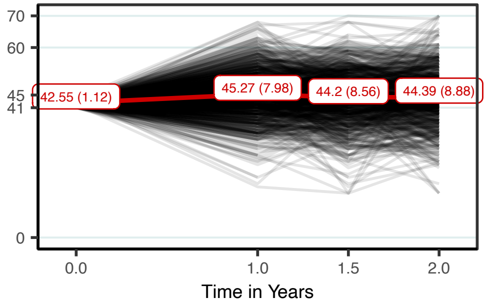

Average Mental Well-being (*n*=4,583)

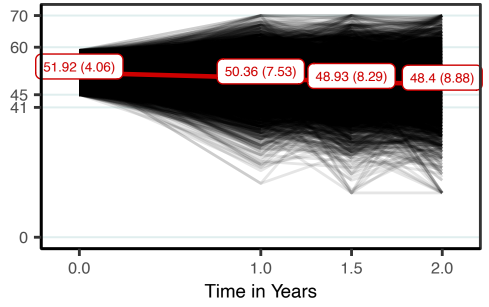

High Well-being (*n*=1,342)

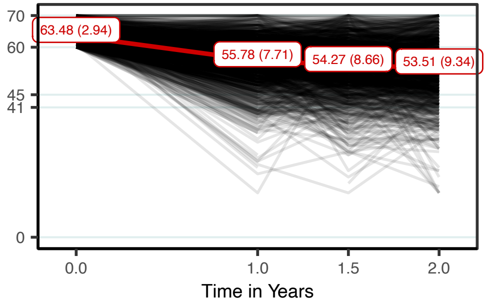

Note: Cut-off scores are based on the official scoring guidelines. Well-being: probable mental health difficulties (0-40); possible mental health difficulties (41-44); average mental well-being (45-59); high well-being (60-70).[27,28]

Table S4 Multivariable Analyses (Adjusted for Cohort, Allocation, and Multiple Comparisons) of Repeated Associations for Girls based on the Three-Level Random Intercept Model

	Depression, CES-D			Social-Emotional-Behavioural Difficulties, SDQ			Well-being, WEMWBS		
	coefficient	95% CI	*p*	coefficient	95% CI	*p*	coefficient	95% CI	*p*
**Model 1: Student-level + School context/ community/ operational features (except student-rated school climate)**									
Student-level age†	0.90	[0.39, 1.42]	**0.001***	0.79	[0.43, 1.14]	**<0.001***	-1.47	[-1.98, -0.96]	**<0.001***
Time(T1)*Age	NA	NA	NA	-0.38	[-0.67, -0.09]	**0.011***	0.23	[-0.26, 0.73]	0.355
Time(T2)*Age	NA	NA	NA	-0.43	[-0.73, -0.13]	**0.005***	0.90	[0.39, 1.40]	**<0.001***
Time(T3)*Age	NA	NA	NA	-0.66	[-0.96, -0.35]	**<0.001***	0.67	[0.16, 1.18]	**0.010***
Ethnicity(White)	NA	NA	NA	0.73	[0.22, 1.23]	**0.005***	NA	NA	NA
Time(T1)*Ethnicity	NA	NA	NA	0.28	[-0.09, 0.66]	0.135	NA	NA	NA
Time(T2)*Ethnicity	NA	NA	NA	0.53	[0.15, 0.91]	**0.006***	NA	NA	NA
Time(T3)*Ethnicity	NA	NA	NA	0.43	[0.04, 0.81]	**0.031**	NA	NA	NA
% Free school meals	NA	NA	NA	0.03	[-0.01, 0.07]	0.160	NA	NA	NA
Time(T1)*Meals	NA	NA	NA	0.02	[-0.01, 0.04]	0.140	NA	NA	NA
Time(T2)*Meals	NA	NA	NA	0.02	[0.00, 0.05]	0.085	NA	NA	NA
Time(T3)*Meals	NA	NA	NA	0.05	[0.02, 0.07]	**<0.001***	NA	NA	NA
School-level age	2.23	[0.10, 4.36]	**0.041**	1.57	[0.43, 2.71]	**0.007***	-2.36	[-3.87, -0.85]	**0.002***
Time(T1)*Age	2.01	[0.71, 3.30]	**0.002***	NA	NA	NA	NA	NA	NA
Time(T2)*Age	0.39	[-0.92, 1.71]	0.555	NA	NA	NA	NA	NA	NA
Time(T3)*Age	0.15	[-1.17, 1.48]	0.820	NA	NA	NA	NA	NA	NA
School size (per 100 students)	NA	NA	NA	0.02	[-0.09, 0.14]	0.686	-0.05	[-0.20, 0.10]	0.487
Time(T1)*Size	NA	NA	NA	-0.08	[-0.15, -0.02]	**0.012***	0.08	[-0.02, 0.19]	0.117
Time(T2)*Size	NA	NA	NA	-0.05	[-0.11, 0.02]	0.160	0.04	[-0.07, 0.14]	0.513
Time(T3)*Size	NA	NA	NA	-0.04	[-0.11, 0.03]	0.229	0.02	[-0.09, 0.13]	0.676
School quality(Good)	NA	NA	NA	NA	NA	NA	-1.05	[-2.07, -0.03]	**0.043**
School quality(Requires improvement)	NA	NA	NA	NA	NA	NA	-0.91	[-2.37, 0.54]	0.220
Time(T1)*Quality(Good)	NA	NA	NA	NA	NA	NA	0.43	[-0.23, 1.09]	0.198
Time(T2)*Quality(Good)	NA	NA	NA	NA	NA	NA	0.32	[-0.34, 0.99]	0.345
Time(T3)*Quality(Good)	NA	NA	NA	NA	NA	NA	0.14	[-0.53, 0.81]	0.683
Time(T1)*Quality(RI)	NA	NA	NA	NA	NA	NA	0.60	[-0.40, 1.60]	0.238
Time(T2)*Quality(RI)	NA	NA	NA	NA	NA	NA	0.38	[-0.63, 1.39]	0.460
Time(T3)*Quality(RI)	NA	NA	NA	NA	NA	NA	-0.76	[-1.80, 0.28]	0.153
School attainment	-0.03	[-0.07, 0.01]	0.125	-0.02	[-0.05, 0.01]	0.116	NA	NA	NA
Time(T1)*Attainment	0.01	[-0.02, 0.02]	0.866	0.01	[0.00, 0.03]	0.035	NA	NA	NA
Time(T2)*Attainment	-0.01	[-0.02, 0.02]	0.848	0.01	[-0.01, 0.02]	0.447	NA	NA	NA
Time(T3)*Attainment	-0.03	[-0.05, -0.01]	**0.006***	-0.01	[-0.02, 0.01]	0.365	NA	NA	NA
**Model 2: Student-level + School context/ community/ operational features (including student-rated school climate)**									
Age†	0.53	[0.00, 1.06]	**0.049**	0.08	[-0.26, 0.41]	0.646	-0.69	[-1.15, -0.22]	**0.004***
Time(T2)*Age	NA	NA	NA	0.05	[-0.22, 0.31]	0.733	0.36	[-0.09, 0.81]	0.119
Time(T3)*Age	NA	NA	NA	-0.08	[-0.35, 0.19]	0.553	0.09	[-0.37, 0.55]	0.698
Ethnicity(White)	NA	NA	NA	1.11	[0.64, 1.59]	**<0.001***	NA	NA	NA
Time(T2)*Ethnicity	NA	NA	NA	0.30	[-0.04, 0.63]	0.087	NA	NA	NA
Time(T3)*Ethnicity	NA	NA	NA	0.22	[-0.12, 0.56]	0.205	NA	NA	NA
% Free school meals	NA	NA	NA	0.05	[0.01, 0.09]	**0.020**	NA	NA	NA
Time(T2)*Meals	NA	NA	NA	-0.01	[-0.02, 0.02]	0.937	NA	NA	NA
Time(T3)*Meals	NA	NA	NA	0.02	[0.00, 0.05]	0.055	NA	NA	NA

School-Level Age	2.05	[-0.14, 4.24]	0.066	0.71	[-0.39, 1.81]	0.207	-1.19	[-2.63, 0.26]	0.108
Time(T2)*Age	-1.04	[-2.26, 0.17]	0.092	NA	NA	NA	NA	NA	NA
Time(T3)*Age	-1.05	[-2.29, 0.19]	0.097	NA	NA	NA	NA	NA	NA
School size (per 100 students)	NA	NA	NA	-0.04	[-0.15, 0.07]	0.468	-0.01	[-0.14, 0.14]	0.973
Time(T2)*Size	NA	NA	NA	0.04	[-0.02, 0.10]	0.178	-0.06	[-0.16, 0.04]	0.230
Time(T3)*Size	NA	NA	NA	0.04	[-0.02, 0.09]	0.230	-0.05	[-0.15, 0.04]	0.291
School quality$_{\text{(Good)}}$¶	NA	NA	NA	NA	NA	NA	-0.68	[-1.61, 0.25]	0.154
School quality$_{\text{(Requires improvement)}}$¶	NA	NA	NA	NA	NA	NA	-0.02	[-1.37, 1.33]	0.976
Time(T2)*Quality(Good)	NA	NA	NA	NA	NA	NA	-0.04	[-0.63, 0.56]	0.901
Time(T3)*Quality(Good)	NA	NA	NA	NA	NA	NA	-0.10	[-0.70, 0.50]	0.737
Time(T2)*Quality(RI)	NA	NA	NA	NA	NA	NA	-0.16	[-1.08, 0.75]	0.725
Time(T3)*Quality(RI)	NA	NA	NA	NA	NA	NA	-1.18	[-2.13, -0.23]	**0.015**
School attainment	-0.01	[-0.05, 0.02]	0.474	-0.01	[-0.03, 0.02]	0.788	NA	NA	NA
Time(T2)*Attainment	0.01	[-0.01, 0.03]	0.534	-0.01	[-0.02, 0.01]	0.382	NA	NA	NA
Time(T3)*Attainment	-0.02	[-0.04, 0.00]	0.120	-0.01	[-0.02, 0.00]	0.051	NA	NA	NA
Student-rated school climate†‡ (Student-level)	-4.80	[-5.09, -4.51]	**<0.001***	-2.60	[-2.75, -2.45]	**<0.001***	4.46	[4.13, 4.80]	**<0.001***
Time(T2)*ClimateStudent	NA	NA	NA	NA	NA	NA	-0.30	[-0.71, 0.11]	0.157
Time(T3)*ClimateStudent	NA	NA	NA	NA	NA	NA	-0.32	[-0.74, 0.10]	0.137
Student-rated school climate‡ (School-level)	-6.82	[-8.50, -5.14]	**<0.001***	-2.79	[-3.62, -1.97]	**<0.001***	5.06	[3.69, 6.42]	**<0.001***

Note: The baseline assessment (T0) is used as the reference timepoint, except for student-rated school climate where only data was collected at T1, T2 and T3. Hence, model 2 only includes data collected at T1, T2, and T3 and T1 was used as the reference timepoint in model 2. Students are nested within schools. NA was used to indicate that associations were not tested because they were non-significant in univariable analyses. Given the small numbers observed in our sample and to facilitate data analyses, we coded 'Ethnicity' as 'White' and 'other ethnic groups' (incl. Arab, Asian, Black/African/Caribbean, mixed ethnic groups, other ethnic groups). CES-D = Center for Epidemiological Studies-Depression. RI = requires improvement. SDQ = Strengths and Difficulties Questionnaire. WEMWBS = Warwick-Edinburgh Mental Well-Being Scale.

† cluster (school-level) centred.

‡ time varying.

¶ reference: school quality = outstanding.

* p < .05 (after adjustment for multiple comparisons; Benjamini-Hochberg correction).

Table S5. Multivariable Analyses (Adjusted for Cohort, Allocation, and Multiple Comparisons) of Repeated Associations for Boys based on the Three-Level Random Intercept Model

	Depression, CES-D			Social-Emotional-Behavioural Difficulties, SDQ			Well-being, WEMWBS		
	**coefficient**	**95% CI**	***p***	**coefficient**	**95% CI**	***p***	**coefficient**	**95% CI**	***p***
**Model 1: Student-level + School context/ community/ operational features (except student-rated school climate)**									
Ethnicity(White)	NA	NA	NA	0.53	[-0.01, 1.07]	0.054	NA	NA	NA
Urbanicity(Rural)	NA	NA	NA	-1.16	[-1.95, -0.37]	**0.004***	NA	NA	NA
% Free school meals	NA	NA	NA	NA	NA	NA	NA	NA	NA
% School ethnicity(=White)	NA	NA	NA	0.02	[0.00, 0.03]	0.061	NA	NA	NA
Time(T1)*SchoolEthnicity	NA	NA	NA	-0.01	[-0.02, 0.00]	0.060	NA	NA	NA
Time(T2)*SchoolEthnicity	NA	NA	NA	-0.01	[-0.02, 0.00]	**0.009***	NA	NA	NA
Time(T3)*SchoolEthnicity	NA	NA	NA	-0.02	[-0.03, -0.01]	**0.004***	NA	NA	NA
Teacher-rated school climate‡	-0.64	[-1.27, -0.02]	**0.043**	NA	NA	NA	NA	NA	NA
**Model 2: Student-level + School context/ community/ operational features (including student-rated school climate)**									
Ethnicity(White)	NA	NA	NA	0.57	[0.04, 1.10]	**0.036**	NA	NA	NA
Urbanicity(Rural)	NA	NA	NA	-1.19	[-1.88, -0.49]	**0.001***	NA	NA	NA
% School ethnicity(=White)	NA	NA	NA	0.01	[0.00, 0.03]	0.167	NA	NA	NA
Time(T2)*SchoolEthnicity	NA	NA	NA	-0.01	[-0.02, 0.00]	0.067	NA	NA	NA
Time(T3)*SchoolEthnicity	NA	NA	NA	-0.01	[-0.02, 0.00]	0.074	NA	NA	NA
Teacher-rated school climate‡	-0.77	[-1.45, -0.10]	**0.025**	NA	NA	NA	NA	NA	NA
Student-rated school climate†‡ (Student-level)	-3.46	[-3.71, -3.21]	**<0.001***	-2.30	[-2.46, -2.14]	**<0.001***	3.37	[3.13, 3.61]	**<0.001***
Student-rated school climate‡ (School-level)	-3.86	[-4.99, -2.74]	**<0.001***	-2.47	[-3.22, -1.71]	**<0.001***	3.38	[2.24, 4.52]	**<0.001***

Note: The baseline assessment (T0) is used as the reference timepoint, except for student-rated school climate where only data was collected at T1, T2 and T3. Hence, model 2 only includes data collected at T1, T2, and T3 and T1 was used as the reference timepoint in model 2. Students are nested within schools. NA was used to indicate that associations were not tested because they were non-significant in univariable analyses. Given the small numbers observed in our sample and to facilitate data analyses, we coded 'Ethnicity' as 'White' and 'other ethnic groups' (incl. Arab, Asian, Black/African/Caribbean, mixed ethnic groups, other ethnic groups). CES-D = Center for Epidemiological Studies-Depression; SDQ = Strengths and Difficulties Questionnaire; WEMWBS = Warwick-Edinburgh Mental Well-Being Scale.
† cluster (school-level) centred.
‡ time varying.
* $p < .05$ (after adjustment for multiple comparisons; Benjamini-Hochberg correction).

Table S6 Univariable Analyses (Unadjusted) of Repeated Associations based on the Three-Level Random Intercept Model

	Depression, CES-D				Social-Emotional-Behavioural Difficulties, SDQ				Well-being, WEMWBS			
	Unadjusted estimates			LRT	Unadjusted estimates			LRT	Unadjusted estimates			LRT
	**coefficient**	**95% CI**	***p***	***p***	**coefficient**	**95% CI**	***p***	***p***	**coefficient**	**95% CI**	***p***	***p***
**Student-level (level 2)**												
Demographics												
Student-Level Age†	0.41	[0.03, 0.78]	**0.032**	0.696	0.41	[0.14, 0.68]	**0.003**	**0.003**	-0.87	[-1.25, -0.49]	**<0.001**	**0.002**
Time(T1)*Age	NA	NA	NA		-0.31	[-0.53, -0.08]	**0.007**		0.08	[-0.29, 0.45]	0.663	
Time(T2)*Age	NA	NA	NA		-0.34	[-0.57, -0.12]	**0.003**		0.54	[0.18, 0.91]	**0.004**	
Time(T3)*Age	NA	NA	NA		-0.38	[-0.61, -0.16]	**0.001**		0.54	[0.17, 0.91]	**0.004**	
Gender(Female)	1.99	[1.44, 2.53]	**<0.001**	**< 0.001**	0.26	[-0.07, 0.60]	0.125	**< 0.001**	-1.28	[-1.75, -0.81]	**<0.001**	**<0.001**
Time(T1)*Gender	2.67	[2.19, 3.15]	**<0.001**		1.29	[1.03, 1.56]	**<0.001**		-1.49	[-1.93, -1.05]	**<0.001**	
Time(T2)*Gender	3.98	[3.50, 4.46]	**<0.001**		1.84	[1.57, 2.10]	**<0.001**		-2.72	[-3.15, -2.28]	**<0.001**	
Time(T3)*Gender	4.24	[3.76, 4.72]	**<0.001**		1.90	[1.63, 2.16]	**<0.001**		-2.94	[-3.38, -2.51]	**<0.001**	
Ethnicity(White)	0.09	[-0.49, 0.67]	0.764	0.875	0.78	[0.41, 1.14]	**<0.001**	0.995	-0.33	[-0.81, 0.15]	0.179	0.195
Time(T1)*Ethnicity	NA	NA	NA		NA	NA	NA		NA	NA	NA	
Time(T2)*Ethnicity	NA	NA	NA		NA	NA	NA		NA	NA	NA	
Time(T3)*Ethnicity	NA	NA	NA		NA	NA	NA		NA	NA	NA	
Student-Level School Climate												
Student-Rated School Climate†‡	-4.18	[-4.37, -3.99]	**<0.001**	0.375	-2.44	[-2.54, -2.34]	**<0.001**	0.736	3.87	[3.71, 4.03]	**<0.001**	0.057
Time(T2)*Climate	NA	NA	NA		NA	NA	NA		NA	NA	NA	
Time(T3)*Climate	NA	NA	NA		NA	NA	NA		NA	NA	NA	
**School-level (level 3)**												
School context												
Urbanicity(Rural)	-1.23	[-2.28, -0.19]	**0.021**	0.320	-0.87	[-1.52, -0.22]	**0.009**	0.149	0.88	[0.01, 1.76]	**0.048**	0.140
Time(T1)* Urbanicity	NA	NA	NA		NA	NA	NA		NA	NA	NA	
Time(T2)* Urbanicity	NA	NA	NA		NA	NA	NA		NA	NA	NA	
Time(T3)* Urbanicity	NA	NA	NA		NA	NA	NA		NA	NA	NA	
Area Deprivation	-0.06	[-0.20, 0.09]	0.440	0.752	-0.07	[-0.16, 0.02]	0.110	0.667	0.01	[-0.12, 0.12]	0.988	0.221
Time(T1)*Deprivation	NA	NA	NA		NA	NA	NA		NA	NA	NA	
Time(T2)*Deprivation	NA	NA	NA		NA	NA	NA		NA	NA	NA	
Time(T3)*Deprivation	NA	NA	NA		NA	NA	NA		NA	NA	NA	
School community												
% Free school meals	-0.01	[-0.04, 0.04]	0.952	0.063	0.01	[-0.02, 0.04]	0.600	**0.008**	0.02	[-0.02, 0.06]	0.292	0.557
Time(T1)*Meals	NA	NA	NA		0.01	[-0.01, 0.02]	0.256		NA	NA	NA	
Time(T2)*Meals	NA	NA	NA		0.02	[0.00, 0.03]	**0.046**		NA	NA	NA	
Time(T3)*Meals	NA	NA	NA		0.03	[0.01, 0.04]	**0.001**		NA	NA	NA	
% SEND support†	-0.02	[-0.10, 0.06]	0.636	0.368	0.02	[-0.03, 0.07]	0.405	0.648	0.02	[-0.04, 0.08]	0.515	0.760
Time(T1)*SEND	NA	NA	NA		NA	NA	NA		NA	NA	NA	
Time(T2)*SEND	NA	NA	NA		NA	NA	NA		NA	NA	NA	
Time(T3)*SEND	NA	NA	NA		NA	NA	NA		NA	NA	NA	
% School ethnicity(=White)	-0.01	[-0.02, 0.02]	0.891	**0.016**	0.01	[0.00, 0.01]	0.539	0.050	-0.01	[-0.03, 0.00]	0.068	**0.001**
Time(T1)*SchoolEthnicity	-0.01	[-0.02, 0.00]	0.104		NA	NA	NA		0.01	[0.00, 0.02]	**0.007**	
Time(T2)*SchoolEthnicity	-0.01	[-0.03, 0.00]	**0.004**		NA	NA	NA		0.02	[0.01, 0.03]	**<0.001**	
Time(T3)*SchoolEthnicity	-0.01	[-0.02, 0.00]	**0.007**		NA	NA	NA		0.01	[0.01, 0.02]	**0.002**	

School-Level Age	0.45	[-1.27, 2.16]	0.611¥	**<0.001**	1.31	[0.22, 2.40]	**0.019**	**< 0.001**	-1.02	[-2.32, 0.29]	0.128¥	0.154
Time(T1)*Age	1.25	[0.29, 2.22]	**0.011¥**		-0.17	[-0.70, 0.35]	0.521		NA	NA	NA	
Time(T2)*Age	-0.35	[-1.33, 0.63]	0.487¥		-0.98	[-1.52, -0.45]	**<0.001**		NA	NA	NA	
Time(T3)*Age	-0.84	[-1.82, 0.15]	0.097¥		-0.92	[-1.47, -0.38]	**0.001**		NA	NA	NA	
Operational features												
School size per 100 students	-0.08	[-0.19, 0.04]	0.210	0.051	-0.01	[-0.09, 0.07]	0.781	**0.001**	-0.01	[-0.10, 0.10]	0.929	0.118
Time(T1)*Size	NA	NA	NA		-0.05	[-0.10, -0.01]	**0.009**		NA	NA	NA	
Time(T2)*Size	NA	NA	NA		-0.05	[-0.09, -0.01]	**0.026**		NA	NA	NA	
Time(T3)*Size	NA	NA	NA		-0.08	[-0.12, -0.04]	**<0.001**		NA	NA	NA	
School sex(Female)	0.12	[-1.04, 1.29]	0.839	**<0.001**	-0.50	[-1.24, 0.25]	0.191	**<0.001**	0.39	[-0.62, 1.39]	0.449	**<0.001**
Time(T1)*Sex	0.98	[0.32, 1.64]	**0.004**		0.49	[0.13, 0.85]	**0.008**		-0.81	[-1.40, -0.21]	**0.008**	
Time(T2)*Sex	2.20	[1.54, 2.86]	**<0.001**		1.02	[0.66, 1.39]	**<0.001**		-1.74	[-2.34, -1.14]	**<0.001**	
Time(T3)*Sex	2.38	[1.72, 3.04]	**<0.001**		0.81	[0.44, 1.17]	**<0.001**		-1.79	[-2.39, -1.19]	**<0.001**	
Student:teacher ratio	-0.07	[-0.30, 0.15]	0.517	0.515	-0.08	[-0.22, 0.06]	0.242	0.935	0.02	[-0.16, 0.20]	0.850	0.804
Time(T1)*Ratio	NA	NA	NA		NA	NA	NA		NA	NA	NA	
Time(T2)*Ratio	NA	NA	NA		NA	NA	NA		NA	NA	NA	
Time(T3)*Ratio	NA	NA	NA		NA	NA	NA		NA	NA	NA	
School quality(Good)¶	-0.10	[-1.19, 0.98]	0.850	**0.015**	0.37	[-0.30, 1.04]	0.283	**0.048**	-0.34	[-1.25, 0.57]	0.463	**<0.001**
School quality(Requires improvement)¶	-0.35	[-1.85, 1.16]	0.652		0.63	[-0.30, 1.56]	0.185		0.06	[-1.20, 1.32]	0.925	
Time(T1)*Quality(Good)	-0.94	[-1.55, -0.34]	**0.002**		-0.38	[-0.71, -0.05]	**0.025**		0.68	[0.14, 1.23]	**0.014**	
Time(T2)*Quality(Good)	-0.85	[-1.46, -0.25]	**0.006**		-0.39	[-0.72, -0.05]	**0.023**		1.02	[0.47, 1.57]	**<0.001**	
Time(T3)*Quality(Good)	-0.75	[-1.36, -0.15]	**0.015**		-0.26	[-0.59, 0.08]	0.129		0.90	[0.35, 1.45]	**0.001**	
Time(T1)*Quality(RI)	-0.68	[-1.57, 0.20]	0.131		-0.69	[-1.18, -0.20]	**0.006**		0.92	[0.12, 1.72]	**0.024**	
Time(T2)*Quality(RI)	-0.70	[-1.59, 0.18]	0.120		-0.74	[-1.23, -0.25]	**0.003**		0.98	[0.18, 1.78]	**0.017**	
Time(T3)*Quality(RI)	0.06	[-0.82, 0.95]	0.889		-0.36	[-0.85, 0.13]	0.147		-0.05	[-0.85, 0.74]	0.894	
SEL provision	0.05	[-0.10, 0.21]	0.507	0.056	0.02	[-0.08, 0.11]	0.730	0.552	-0.08	[-0.20, 0.05]	0.249	0.188
Time(T1)*SEL	NA	NA	NA		NA	NA	NA		NA	NA	NA	
Time(T2)*SEL	NA	NA	NA		NA	NA	NA		NA	NA	NA	
Time(T3)*SEL	NA	NA	NA		NA	NA	NA		NA	NA	NA	
School attainment	-0.01	[-0.04, 0.02]	0.646	**0.037**	-0.01	[-0.03, 0.01]	0.168	0.266	-0.01	[-0.03, 0.02]	0.807	0.783
Time(T1)*Attainment	0.02	[0.00, 0.03]	0.060		NA	NA	NA		NA	NA	NA	
Time(T2)*Attainment	0.01	[0.00, 0.03]	0.138		NA	NA	NA		NA	NA	NA	
Time(T3)*Attainment	-0.01	[-0.02, 0.01]	0.533		NA	NA	NA		NA	NA	NA	
Teacher-Rated School Climate‡	-1.35	[-2.09, -0.60]	**<0.001**	**0.006**	-0.73	[-1.14, -0.32]	**0.001**	**0.004**	0.95	[0.29, 1.62]	**0.005**	**0.001**
Time(T1)*Climate	0.79	[0.05, 1.53]	**0.037**		0.44	[0.04, 0.85]	**0.033**		-0.65	[-1.32, 0.03]	**0.060**	
Time(T2)*Climate	1.34	[0.60, 2.08]	**<0.001**		0.67	[0.27, 1.08]	**0.001**		-1.33	[-2.00, -0.66]	**<0.001**	
Time(T3)*Climate	0.79	[0.01, 1.57]	**0.047**		0.70	[0.28, 1.13]	**0.001**		-0.63	[-1.33, 0.08]	0.082	
Student-Rated School Climate‡	-6.48	[-7.65, -5.30]	**<0.001**	0.121	-2.75	[-3.40, -2.10]	**<0.001**	0.497	4.47	[3.46, 5.47]	**<0.001**	0.317
Time(T2)*Climate	NA	NA	NA		NA	NA	NA		NA	NA	NA	
Time(T3)*Climate	NA	NA	NA		NA	NA	NA		NA	NA	NA	

Note: The baseline assessment (T0) is used as the reference group, except for student-rated school climate where only data was collected at T1, T2 and T3. Hence, for student-rated school climate T1 was used as the reference group. Students are nested within schools. Given the small numbers observed in our sample and to facilitate data analyses, we coded ‘Ethnicity’ as ‘White’ and ‘other ethnic groups’ (incl. Arab, Asian, Black/African/Caribbean, mixed ethnic groups, other ethnic groups). CES-D = Center for Epidemiological Studies-Depression; LRT = likelihood ratio test to test whether the time-by-factor interaction term needs to be included in the model; NA = only the main effect of time, but not the time-by-factor interaction term, was included in the model as the LRT was non-significant; RI = requires improvement; SDQ = Strengths and Difficulties Questionnaire; SEL = social and emotional learning; SEND = special educational needs and disability; WEMWBS = Warwick-Edinburgh Mental Well-Being Scale.

† cluster (school-level) centred.
‡ time varying.
¥ To address convergence problems, due to high tolerance values, this model was estimated with the optimiser “bobyqa” for a quadratic approximation.
¶ reference: school quality = outstanding.

Table S7 Univariable Analyses (Unadjusted) of Repeated Associations for Girls based on the Three-Level Random Intercept Model

	Depression, CES-D				Social-Emotional-Behavioural Difficulties, SDQ				Well-being, WEMWBS			
	Unadjusted estimates			LRT¶	Unadjusted estimates			LRT¶	Unadjusted estimates			LRT¶
	coefficient	95% CI	*p*	*p*	coefficient	95% CI	*p*	*p*	coefficient	95% CI	*p*	*p*
**Student-level (level 2)** ***Demographics***												
Student-Level Age†	0.80	[0.28, 1.33]	**0.003**	0.765	0.58	[0.21, 0.95]	**0.002**	**0.002**	-1.42	[-1.94, -0.90]	**<0.001**	**0.014**
Time(T1)*Age	NA	NA	NA		-0.32	[-0.61, -0.02]	**0.037**		0.22	[-0.27, 0.72]	0.382	
Time(T2)*Age	NA	NA	NA		-0.43	[-0.73, -0.13]	**0.004**		0.73	[0.23, 1.23]	**0.004**	
Time(T3)*Age	NA	NA	NA		-0.56	[-0.85, -0.26]	**<0.001**		0.61	[0.11, 1.10]	**0.017**	
Ethnicity$_{(White)}$	0.47	[-0.33, 1.28]	0.251	0.265	0.63	[0.09, 1.17]	**0.023**	**0.025**	-0.37	[-1.01, 0.27]	0.255	0.144
Time(T1)*Ethnicity	NA	NA	NA		0.31	[-0.08, 0.69]	0.119		NA	NA	NA	
Time(T2)*Ethnicity	NA	NA	NA		0.58	[0.20, 0.97]	**0.003**		NA	NA	NA	
Time(T3)*Ethnicity	NA	NA	NA		0.42	[0.03, 0.80]	**0.034**		NA	NA	NA	
Student-Level School Climate												
Student-Rated School Climate†‡	-4.85	[-5.12, -4.57]	**<0.001**	0.999	-2.58	[-2.72, -2.44]	**<0.001**	0.104	4.59	[4.28, 4.90]	**<0.001**	**0.037**
Time(T2)*Climate	NA	NA	NA		NA	NA	NA		-0.45	[-0.83, -0.07]	**0.021**	
Time(T3)*Climate	NA	NA	NA		NA	NA	NA		-0.42	[-0.81, -0.03]	**0.033**	
**School-level (level 3)** ***School context***												
Urbanicity$_{(Rural)}$	-1.29	[-2.79, 0.21]	0.092	0.206	-0.74	[-1.66, 0.18]	0.115	0.386	0.96	[-0.24, 2.16]	0.117	0.207
Time(T1)* Urbanicity	NA	NA	NA		NA	NA	NA		NA	NA	NA	
Time(T2)* Urbanicity	NA	NA	NA		NA	NA	NA		NA	NA	NA	
Time(T3)* Urbanicity	NA	NA	NA		NA	NA	NA		NA	NA	NA	
Area Deprivation	-0.03	[-0.24, 0.18]	0.775	0.619	-0.08	[-0.20, 0.05]	0.233	0.535	0.02	[-0.14, 0.19]	0.782	0.070
Time(T1)*Deprivation	NA	NA	NA		NA	NA	NA		NA	NA	NA	
Time(T2)*Deprivation	NA	NA	NA		NA	NA	NA		NA	NA	NA	
Time(T3)*Deprivation	NA	NA	NA		NA	NA	NA		NA	NA	NA	
School community												
% Free school meals	0.02	[-0.04, 0.08]	0.488	0.065	0.02	[-0.02, 0.06]	0.276	**<0.001**	-0.01	[-0.06, 0.04]	0.701	0.270
Time(T1)*Meals	NA	NA	NA		0.01	[-0.01, 0.03]	0.324		NA	NA	NA	
Time(T2)*Meals	NA	NA	NA		0.02	[0.00, 0.04]	0.080		NA	NA	NA	
Time(T3)*Meals	NA	NA	NA		0.05	[0.03, 0.07]	**< 0.001**		NA	NA	NA	
% SEND support†	0.05	[-0.07, 0.17]	0.404	0.373	0.07	[0.00, 0.13]	0.067	0.063	-0.03	[-0.12, 0.06]	0.500	0.304
Time(T1)*SEND	NA	NA	NA		NA	NA	NA		NA	NA	NA	
Time(T2)*SEND	NA	NA	NA		NA	NA	NA		NA	NA	NA	
Time(T3)*SEND	NA	NA	NA		NA	NA	NA		NA	NA	NA	
% School ethnicity(=White)	0.01	[-0.02, 0.03]	0.700	0.963	0.01	[-0.01, 0.02]	0.371	0.385	-0.01	[-0.03, 0.01]	0.391	0.304
Time(T1)*SchoolEthnicity	NA	NA	NA		NA	NA	NA		NA	NA	NA	
Time(T2)*SchoolEthnicity	NA	NA	NA		NA	NA	NA		NA	NA	NA	
Time(T3)*SchoolEthnicity	NA	NA	NA		NA	NA	NA		NA	NA	NA	
School-Level Age	2.02	[-0.17,4.20]	0.071¥	**0.003**	1.66	[0.36, 2.96]	**0.012**	0.080	-2.36	[-3.99, -0.73]	**0.005**	0.682
Time(T1)*Age	2.00	[0.75,3.24]	**0.002¥**		NA	NA	NA		NA	NA	NA	
Time(T2)*Age	0.36	[-0.90,1.63]	0.572¥		NA	NA	NA		NA	NA	NA	
Time(T3)*Age	-0.12	[-1.40,1.15]	0.849¥		NA	NA	NA		NA	NA	NA	

Operational features												
School size per 100 students	-0.12	[-0.29, 0.05]	0.153	0.101	-0.05	[-0.16, 0.06]	0.396	**0.007**	-0.06	[-0.21, 0.09]	0.423	**0.012**
Time(T1)*Size	NA	NA	NA		-0.04	[-0.10, 0.02]	0.162		0.12	[0.03, 0.21]	**0.011**	
Time(T2)*Size	NA	NA	NA		-0.04	[-0.10, 0.01]	0.128		0.12	[0.03, 0.21]	**0.011**	
Time(T3)*Size	NA	NA	NA		-0.10	[-0.15, -0.04]	**0.001**		0.14	[0.05, 0.23]	**0.003**	
School sex(Female)	-0.90	[-2.38, 0.58]	0.235	0.236	-0.74	[-1.64, 0.15]	0.104	0.326	0.93	[-0.25, 2.10]	0.122	0.623
Time(T1)*Sex	NA	NA	NA		NA	NA	NA		NA	NA	NA	
Time(T2)*Sex	NA	NA	NA		NA	NA	NA		NA	NA	NA	
Time(T3)*Sex	NA	NA	NA		NA	NA	NA		NA	NA	NA	
Student:teacher ratio	-0.05	[-0.38, 0.29]	0.793	0.074	-0.08	[-0.28, 0.12]	0.445	0.628	-0.04	[-0.31, 0.22]	0.749	0.241
Time(T1)*Ratio	NA	NA	NA		NA	NA	NA		NA	NA	NA	
Time(T2)*Ratio	NA	NA	NA		NA	NA	NA		NA	NA	NA	
Time(T3)*Ratio	NA	NA	NA		NA	NA	NA		NA	NA	NA	
School quality(Good)¶	0.14	[-1.28, 1.56]	0.849	0.201	0.36	[-0.48, 1.21]	0.399	0.329	-0.62	[-1.79, 0.55]	0.299	**0.022**
School quality(Requires improvement)¶	0.38	[-1.63, 2.39]	0.710		0.76	[-0.44, 1.96]	0.213		-0.08	[-1.77, 1.60]	0.922	
Time(T1)*Quality(Good)	NA	NA	NA		NA	NA	NA		0.35	[-0.33, 1.03]	0.311	
Time(T2)*Quality(Good)	NA	NA	NA		NA	NA	NA		0.25	[-0.43, 0.93]	0.474	
Time(T3)*Quality(Good)	NA	NA	NA		NA	NA	NA		0.18	[-0.50, 0.85]	0.611	
Time(T1)*Quality(RI)	NA	NA	NA		NA	NA	NA		0.41	[-0.67, 1.49]	0.461	
Time(T2)*Quality(RI)	NA	NA	NA		NA	NA	NA		-0.03	[-1.11, 1.05]	0.954	
Time(T3)*Quality(RI)	NA	NA	NA		NA	NA	NA		-1.38	[-2.47, -0.30]	**0.012**	
SEL provision	0.05	[-0.16, 0.27]	0.628	0.080	0.02	[-0.12, 0.15]	0.808	0.115	-0.05	[-0.23, 0.12]	0.544	0.116
Time(T1)*SEL	NA	NA	NA		NA	NA	NA		NA	NA	NA	
Time(T2)*SEL	NA	NA	NA		NA	NA	NA		NA	NA	NA	
Time(T3)*SEL	NA	NA	NA		NA	NA	NA		NA	NA	NA	
School attainment	-0.02	[-0.06, 0.03]	0.448	**0.007**	-0.02	[-0.04, 0.01]	0.221	**0.002**	0.02	[-0.02, 0.05]	0.343	0.261
Time(T1)*Attainment	0.01	[-0.02, 0.03]	0.556		0.01	[-0.01, 0.01]	0.677		NA	NA	NA	
Time(T2)*Attainment	-0.01	[-0.02, 0.02]	0.863		-0.01	[-0.02, 0.01]	0.532		NA	NA	NA	
Time(T3)*Attainment	-0.03	[-0.05, -0.01]	**0.009**		-0.02	[-0.03, -0.01]	**0.002**		NA	NA	NA	
Teacher-Rated School Climate‡	-0.54	[-1.25, 0.17]	0.134	0.514	-0.23	[-0.61, 0.14]	0.226¥	0.347	0.44	[-0.16, 1.05]	0.149	0.082
Time(T1)*Climate	NA	NA	NA		NA	NA	NA		NA	NA	NA	
Time(T2)*Climate	NA	NA	NA		NA	NA	NA		NA	NA	NA	
Time(T3)*Climate	NA	NA	NA		NA	NA	NA		NA	NA	NA	
Student-Rated School Climate‡	-7.31	[-8.82, -5.80]	**<0.001**	0.510	-2.98	[-3.76, -2.20]	**< 0.001**	0.684	5.31	[4.09, 6.54]	**<0.001**	0.926
Time(T2)*Climate	NA	NA	NA		NA	NA	NA		NA	NA	NA	
Time(T3)*Climate	NA	NA	NA		NA	NA	NA		NA	NA	NA	

Note: The baseline assessment (T0) is used as the reference group, except for student-rated school climate where only data was collected at T1, T2 and T3. Hence, for student-rated school climate T1 was used as the reference group. Students are nested within schools. Given the small numbers observed in our sample and to facilitate data analyses, we coded 'Ethnicity' as 'White' and 'other ethnic groups' (incl. Arab, Asian, Black/African/Caribbean, mixed ethnic groups, other ethnic groups). CES-D = Center for Epidemiological Studies-Depression; LRT = likelihood ratio test to test whether the time-by-factor interaction term needs to be included in the model; NA = only the main effect of time, but not the time-by-factor interaction term, was included in the model as the LRT was non-significant. RI = requires improvement; SDQ = Strengths and Difficulties Questionnaire; SEL = social and emotional learning; SEND = special educational needs and disability; WEMWBS = Warwick-Edinburgh Mental Well-Being Scale.

† cluster (school-level) centred.

‡ time varying.

¥ To address convergence problems, due to high tolerance values, this model was estimated with the optimiser "bobyqa" for a quadratic approximation.

¶ reference: school quality = outstanding.

Table S8 Univariable Analyses (Unadjusted) of Repeated Associations for Boys based on the Three-Level Random Intercept Model

	Depression, CES-D				Social-Emotional-Behavioural Difficulties, SDQ				Well-being, WEMWBS			
	Unadjusted estimates			LRT¶	Unadjusted estimates			LRT¶	Unadjusted estimates			LRT¶
	**coefficient**	**95% CI**	***p***	***p***	**coefficient**	**95% CI**	***p***	***p***	**coefficient**	**95% CI**	***p***	***p***
**Student-level (level 2)** ***Demographics***												
Student-Level Age†	-0.04	[-0.53, 0.45]	0.886	0.957	0.03	[-0.32, 0.38]	0.873	0.616	-0.06	[-0.52, 0.39]	0.787	0.276
Time(T1)*Age	NA	NA	NA		NA	NA	NA		NA	NA	NA	
Time(T2)*Age	NA	NA	NA		NA	NA	NA		NA	NA	NA	
Time(T3)*Age	NA	NA	NA		NA	NA	NA		NA	NA	NA	
Ethnicity(White)	-0.01	[-0.76, 0.76]	0.999	0.636	0.70	[0.16, 1.24]	**0.012**	0.561	-0.65	[-1.37, 0.06]	0.073	0.622
Time(T1)*Ethnicity	NA	NA	NA		NA	NA	NA		NA	NA	NA	
Time(T2)*Ethnicity	NA	NA	NA		NA	NA	NA		NA	NA	NA	
Time(T3)*Ethnicity	NA	NA	NA		NA	NA	NA		NA	NA	NA	
Student-Level School Climate												
Student-Rated School Climate†‡	-3.50	[-3.75, -3.24]	**<0.001**	0.065	-2.28	[-2.43, -2.12]	**<0.001**	0.240	3.36	[3.13, 3.60]	**<0.001**	0.696
Time(T2)*Climate	NA	NA	NA		NA	NA	NA		NA	NA	NA	
Time(T3)*Climate	NA	NA	NA		NA	NA	NA		NA	NA	NA	
**School-level (level 3)** ***School context***												
Urbanicity(Rural)	-0.86	[-1.84, 0.12]	0.086	0.938	-0.84	[-1.60, -0.09]	**0.029**	0.486	0.47	[-0.57, 1.51]	0.377	0.651
Time(T1)* Urbanicity	NA	NA	NA		NA	NA	NA		NA	NA	NA	
Time(T2)* Urbanicity	NA	NA	NA		NA	NA	NA		NA	NA	NA	
Time(T3)* Urbanicity	NA	NA	NA		NA	NA	NA		NA	NA	NA	
Area Deprivation	-0.08	[-0.22, 0.05]	0.217	0.723	-0.07	[-0.18, 0.03]	0.172	0.239	-0.02	[-0.17, 0.12]	0.734	0.966
Time(T1)*Deprivation	NA	NA	NA		NA	NA	NA		NA	NA	NA	
Time(T2)*Deprivation	NA	NA	NA		NA	NA	NA		NA	NA	NA	
Time(T3)*Deprivation	NA	NA	NA		NA	NA	NA		NA	NA	NA	
School community												
% Free school meals	0.01	[-0.04, 0.04]	0.974	0.121	0.01	[-0.02, 0.04]	0.631	0.184	0.04	[-0.01, 0.08]	0.101	0.636
Time(T1)*Meals	NA	NA	NA		NA	NA	NA		NA	NA	NA	
Time(T2)*Meals	NA	NA	NA		NA	NA	NA		NA	NA	NA	
Time(T3)*Meals	NA	NA	NA		NA	NA	NA		NA	NA	NA	
% SEND support†	-0.01	[-0.08, 0.07]	0.830	0.063	-0.01	[-0.06, 0.05]	0.928	0.684	0.03	[-0.05, 0.10]	0.459	0.550
Time(T1)*SEND	NA	NA	NA		NA	NA	NA		NA	NA	NA	
Time(T2)*SEND	NA	NA	NA		NA	NA	NA		NA	NA	NA	
Time(T3)*SEND	NA	NA	NA		NA	NA	NA		NA	NA	NA	
% School ethnicity(=White)	-0.01	[-0.03, 0.02]	0.652	0.193	0.02	[0.00, 0.03]	0.098	**0.026**	-0.02	[-0.04, 0.00]	0.078	0.154
Time(T1)*SchoolEthnicity	NA	NA	NA		-0.01	[-0.02, 0.00]	**0.030**		NA	NA	NA	
Time(T2)*SchoolEthnicity	NA	NA	NA		-0.02	[-0.03, 0.00]	**0.006**		NA	NA	NA	
Time(T3)*SchoolEthnicity	NA	NA	NA		-0.01	[-0.03, 0.00]	**0.017**		NA	NA	NA	
School-Level Age	-0.72	[-2.24, 0.81]	0.357¥	0.111	-0.19	[-1.45, 1.06]	0.761	0.186	0.08	[-1.51, 1.66]	0.926	0.488
Time(T1)*Age	NA	NA	NA		NA	NA	NA		NA	NA	NA	
Time(T2)*Age	NA	NA	NA		NA	NA	NA		NA	NA	NA	
Time(T3)*Age	NA	NA	NA		NA	NA	NA		NA	NA	NA	

Operational features												
School size per 100 students	-0.05	[-0.16, 0.07]	0.417	0.674	-0.03	[-0.12, 0.05]	0.459	0.143	-0.02	[-0.14, 0.10]	0.734	0.987
Time(T1)*Size	NA	NA	NA		NA	NA	NA		NA	NA	NA	
Time(T2)*Size	NA	NA	NA		NA	NA	NA		NA	NA	NA	
Time(T3)*Size	NA	NA	NA		NA	NA	NA		NA	NA	NA	
Student:teacher ratio	-0.16	[-0.37, 0.05]	0.144	0.713	-0.11	[-0.27, 0.06]	0.212	0.699	0.10	[-0.11, 0.32]	0.350	0.720
Time(T1)*Ratio	NA	NA	NA		NA	NA	NA		NA	NA	NA	
Time(T2)*Ratio	NA	NA	NA		NA	NA	NA		NA	NA	NA	
Time(T3)*Ratio	NA	NA	NA		NA	NA	NA		NA	NA	NA	
School quality(Good)¶	0.17	[-1.04, 1.39]	0.778	0.192	0.46	[-0.46, 1.37]	0.328	0.475	-0.08	[-1.38, 1.22]	0.905	0.156
School quality(Requires improvement)¶	0.25	[-1.26, 1.76]	0.745		0.36	[-0.77, 1.50]	0.531		0.17	[-1.44, 1.77]	0.840	
Time(T1)*Quality(Good)	NA	NA	NA		NA	NA	NA		NA	NA	NA	
Time(T2)*Quality(Good)	NA	NA	NA		NA	NA	NA		NA	NA	NA	
Time(T3)*Quality(Good)	NA	NA	NA		NA	NA	NA		NA	NA	NA	
Time(T1)*Quality(RI)	NA	NA	NA		NA	NA	NA		NA	NA	NA	
Time(T2)*Quality(RI)	NA	NA	NA		NA	NA	NA		NA	NA	NA	
Time(T3)*Quality(RI)	NA	NA	NA		NA	NA	NA		NA	NA	NA	
SEL provision	0.01	[-0.15, 0.15]	0.980	0.127	0.01	[-0.11, 0.12]	0.909	0.519	-0.08	[-0.23, 0.08]	0.332¥	0.686
Time(T1)*SEL	NA	NA	NA		NA	NA	NA		NA	NA	NA	
Time(T2)*SEL	NA	NA	NA		NA	NA	NA		NA	NA	NA	
Time(T3)*SEL	NA	NA	NA		NA	NA	NA		NA	NA	NA	
School attainment	-0.01	[-0.04, 0.02]	0.427	0.724	-0.02	[-0.04, 0.00]	0.098	0.387	-0.01	[-0.03, 0.03]	0.975	0.212
Time(T1)*Attainment	NA	NA	NA		NA	NA	NA		NA	NA	NA	
Time(T2)*Attainment	NA	NA	NA		NA	NA	NA		NA	NA	NA	
Time(T3)*Attainment	NA	NA	NA		NA	NA	NA		NA	NA	NA	
Teacher-Rated School Climate‡	-0.63	[-1.26, -0.01]	**0.046**	0.803	-0.29	[-0.69, 0.11]	0.159	0.174	-0.12	[-0.74, 0.51]	0.713	0.454
Time(T1)*Climate	NA	NA	NA		NA	NA	NA		NA	NA	NA	
Time(T2)*Climate	NA	NA	NA		NA	NA	NA		NA	NA	NA	
Time(T3)*Climate	NA	NA	NA		NA	NA	NA		NA	NA	NA	
Student-Rated School Climate‡	-3.54	[-4.66, -2.42]	**<0.001**	0.992	-2.02	[-2.76, -1.29]	**<0.001**	0.948	3.04	[1.91, 4.17]	**<0.001**	0.500
Time(T2)*Climate	NA	NA	NA		NA	NA	NA		NA	NA	NA	
Time(T3)*Climate	NA	NA	NA		NA	NA	NA		NA	NA	NA	

Note: The baseline assessment (T0) is used as the reference group, except for student-rated school climate where only data was collected at T1, T2 and T3. Hence, for student-rated school climate T1 was used as the reference group. Students are nested within schools. Given the small numbers observed in our sample and to facilitate data analyses, we coded 'Ethnicity' as 'White' and 'other ethnic groups' (incl. Arab, Asian, Black/African/Caribbean, mixed ethnic groups, other ethnic groups). CES-D = Center for Epidemiological Studies-Depression; LRT = likelihood ratio test to test whether the time-by-factor interaction term needs to be included in the model; NA = only the main effect of time, but not the time-by-factor interaction term, was included in the model as the LRT was non-significant; RI = requires improvement; SDQ = Strengths and Difficulties Questionnaire; SEL = social and emotional learning; SEND = special educational needs and disability; WEMWBS = Warwick-Edinburgh Mental Well-Being Scale.

† cluster (school-level) centred.

‡ time varying.

¥ To address convergence problems, due to high tolerance values, this model was estimated with the optimiser "bobyqa" for a quadratic approximation.

¶ reference: school quality = outstanding.

References for the Supplementary Materials

1. Ford T, Degli Esposti M, Crane C, et al. The Role of Schools in Early Adolescents' Mental Health: Findings From the MYRIAD Study. *J Am Acad Child Adolesc Psychiatry.* 2021;**60**(12):1467–1478. https://doi.org/10.1016/j.jaac.2021.02.016
2. Kuyken W, Nuthall E, Byford S, et al. The effectiveness and cost-effectiveness of a mindfulness training programme in schools compared with normal school provision (MYRIAD): study protocol for a randomised controlled trial. *Trials.* 2017;**18**(1):194. https://doi.org/10.1186/s13063-017-1917-4
3. Kuyken W, Ball S, Crane C, et al. Effectiveness and cost-effectiveness of universal school-based mindfulness training compared with normal school provision in reducing risk of mental health problems and promoting well-being in adolescence: the MYRIAD cluster randomised controlled trial. *Evid Based Ment Health.* 2022;**25**(3):99–109. http://dx.doi.org/10.1136/ebmental-2021-300396
4. Briere FN, Pascal S, Dupere V, Janosz M. School environment and adolescent depressive symptoms: a multilevel longitudinal study. *Pediatrics.* 2013;**131**(3):e702-8. https://doi.org/10.1542/peds.2012-2172
5. Youth in Mind. *British means and standard deviations for the SDQ by age band*. 2023. [accessed 26.04.2021]; Available from: https://www.sdqinfo.org/norms/UKNorm3.pdf.
6. Clarke A, Friede T, Putz R, et al. Warwick-Edinburgh Mental Well-being Scale (WEMWBS): validated for teenage school students in England and Scotland. A mixed methods assessment. *BMC Public Health.* 2011;**11**:487. https://doi.org/10.1186/1471-2458-11-487
7. Wickham H, François R, Henry L, Müller K. *dplyr: A Grammar of Data Manipulation. R package version 1.0.8.* 2022.
8. Harrell FE. *Hmisc: Harrell Miscellaneous. R package version 4.6-0.* 2021.
9. Revelle W. *psych: Procedures for Personality and Psychological Research; R package version 2.1.9.* 2021, Evanston, Illinois, USA: Northwestern University.
10. R Core Team. *R: A language and environment for statistical computing. R version 3.6.2 – "Dark and Stormy Night".* 2019, Vienna, Austria: R Foundation for Statistical Computing.
11. Wickham H, Girlich M. *tidyr: Tidy Messy Data. R package version 1.2.0.* 2022.
12. Yanagida T. *misty: Miscellaneous Functions 'T. Yanagida'. R package version 0.4.3.* 2021.
13. Garbuszus JM, Pfaff B. *readspss: Importing and Exporting SPSS Files. R package version 0.14.* 2021.
14. Wickham H. *ggplot2: Elegant Graphics for Data Analysis*. 2016, New York, USA: Springer-Verlag.
15. Lüdecke D. *sjPlot: Data Visualization for Statistics in Social Science. R package version 2.8.10*. 2021.
16. Bates D, Machler M, Bolker BM, Walker SC. Fitting Linear Mixed-Effects Models Using lme4. *J Stat Softw.* 2015;**67**(1):1–48. https://doi.org/10.18637/jss.v067.i01
17. Fox J, Weisberg S. *An R Companion to Applied Regression, Third Edition*. 2019, Thousand Oaks, CA: Sage.
18. Lüdecke D, Ben-Shachar M, Patil I, Makowski D. Extracting, Computing and Exploring the Parameters of Statistical Models using R. *J Open Source Softw.* 2020;**5**(53):2445. https://doi.org/10.21105/joss.02445
19. Lüdecke D, Ben-Shachar M, Patil I, Waggoner P, Makowski D. performance: An R Package for Assessment, Comparison and Testing of Statistical Models. *J Open Source Softw.* 2021;**6**(60):3139. https://doi.org/10.21105/joss.03139
20. Rosseel Y. lavaan: An R Package for Structural Equation Modeling. *J Stat Softw.* 2012;**48**(2):1–36. https://doi.org/10.18637/jss.v048.i02
21. Torchiano M. *effsize: Efficient Effect Size Computation. R package version 0.8.1*. 2020.
22. Anumendem ND, De Fraine, B, Onghena P, Van Damme J. The impact of coding time on the estimation of school effects. *Qual Quant.* 2013;**47**(2):1021–1040. https://doi.org/10.1007/s11135-011-9581-3
23. Powell MJD. *The BOBYQA algorithm for bound constrained optimization without derivatives*. 2009, Cambridge, England.
24. Radloff LS. The use of the Center for Epidemiologic Studies Depression Scale in adolescents and young adults. *J Youth Adolesc.* 1991;**20**(2):149–66. https://doi.org/10.1007/BF01537606
25. Rushton JL, Forcier M, Schectman RM, Epidemiology of depressive symptoms in the National Longitudinal Study of Adolescent Health. *J Am Acad Child Adolesc Psychiatry.* 2002;**41**(2):199–205. https://doi.org/10.1097/00004583-200202000-00014
26. Goodman R, Renfrew D, Mullick M, Predicting type of psychiatric disorder from Strengths and Difficulties Questionnaire (SDQ) scores in child mental health clinics in London and Dhaka. *Eur Child Adolesc Psychiatry.* 2000;**9**(2):129–34. https://doi.org/10.1007/s007870050008
27. Tennant R, Hiller L, Fishwick R, et al. The Warwick-Edinburgh Mental Well-being Scale (WEMWBS): development and UK validation. *Health Qual Life Outcomes.* 2007;**5**:63. https://doi.org/10.1186/1477-7525-5-63

28. Warwick Medical School. *Collect, score, analyse and interpret WEMWBS*. 2021. [accessed 09.11.2022]; Available from: https://warwick.ac.uk/fac/sci/med/research/platform/wemwbs/using/howto/.
